# From Ethanolamine Precursor Towards ZnO—How N Is Released from the Experimental and Theoretical Points of View

**DOI:** 10.3390/nano9101415

**Published:** 2019-10-03

**Authors:** Alberto Gómez-Núñez, Santiago Alonso-Gil, Concepción López, Pere Roura-Grabulosa, Anna Vilà

**Affiliations:** 1 Department of Electronic and Biomedical Engineering, University of Barcelona, Martí i Franquès 1, 08028 Barcelona, Spain; 2 Institute of Nanoscience and Nanotechnology (IN2UB), University of Barcelona, Joan XXIII s/n, 08028 Barcelona, Spain; 3 FAE Francisco Albero S.A.U., Rafael Barradas 19, Granvia L’Hospitalet Economic District, 08908 L’Hospitalet de Llobregat, Spain; agomez1618@gmail.com; 4 Department of Inorganic and Organic Chemistry, University of Barcelona, Martí i Franquès 1, 08028 Barcelona, Spain; conchi.lopez@qi.ub.es; 5 J. Heyrovský Institute of Physical Chemistry, Czech Academy of Sciences, Dolejškova 2155/3, 18223 Prague 8, Czech Republic; 6 Department of Physics, Campus Montilivi, University of Girona, Edif. PII, 17003 Girona, Spain; pere.roura@udg.cat

**Keywords:** ethanolamine, sol-gel, ZnO precursor, CPMD

## Abstract

This work presents experimental and computational studies on ZnO formation after decomposition of a sol-gel precursor containing ethanolamine and Zn(II) acetate. The structural modifications suffered during decomposition of the monomeric and dimeric Zn(II) complexes formed, containing bidentate deprotonated ethanolamine and acetato ligands, have been described experimentally and explained via Car-Parrinello Molecular Dynamics. Additional metadynamics simulations provide an overview of the dimer evolution by the cleavage of the Zn–N bond, the structural changes produced and their effects on the Zn(II) environment. The results provide conclusive evidence of the relevance of ethanolamine used as a stabilizer in the formation of ZnO.

## 1. Introduction

ZnO-based nanomaterials are crucial in various areas, including biological, optical, photocatalysis, and so forth [1,2,3,4,5,6,7,8,9,10,11,12,13,14]. In general, these nanomaterials are prepared using a zinc(II) salt (usually acetate) and an additional organic ligand to stabilize the precursor [3,4,5,6]. It has also been proven that the purity, morphology and properties of these ZnO nanomaterials depend on several factors (i.e., additional reactants, solvents, temperature, experimental conditions etc.) that affect the final purity [5,6]. The photocatalytic activity of these types of materials, as well as their optical properties, are strongly influenced by the N content [5,6,7,8,9,10,11,12].

Solution-processed ZnO can enable low-cost devices and circuits via mass-production roll-to-roll processes using conventional coating combined with printing techniques (screen and inkjet printing, dip coating, etc.) [6]. Although their performance is usually more limited than that of physical deposition techniques, the morphology and properties of the final product can be tuned through a variety of parameters, making these wet chemical methods very powerful and versatile techniques for ZnO synthesis. In this context, sol-gel based processes are among the methodologies with the greatest expectation for achieving low cost ZnO [1,5,13].

**Figure 1:**
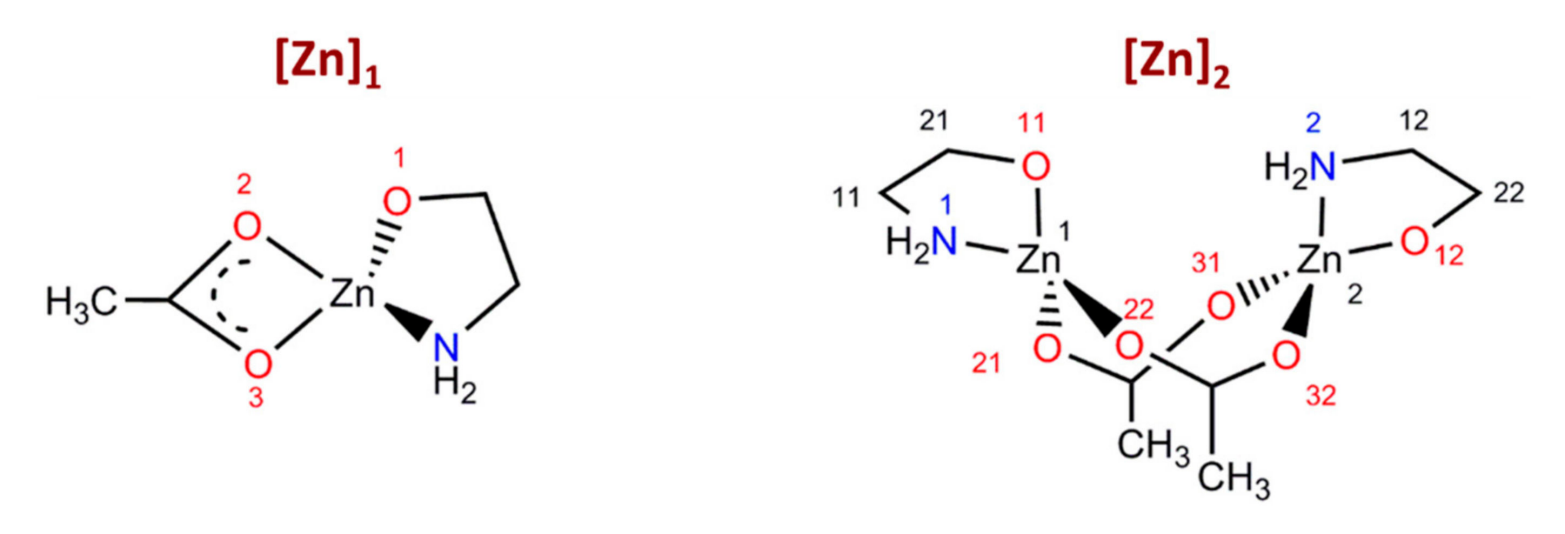
Schematic views of the monomeric and dimeric Zn(II) complexes ([Zn]_1_ and [Zn]_2_, respectively), showing the atom labelling scheme for the computational studies.

It is well known that aminoalcohols, especially ethanolamine (EA), are by far the organic ligands most widely used as stabilizers. Previous studies have revealed that—(a) the tetrameric complex, [Zn]_4_, formed in the reaction of zinc acetate dihydrate (ZAD) with EA is clearly more stable in solid than in solution, where monomeric and dimeric species ([Zn]_1_ and [Zn]_2_, respectively, shown in Figure 1) prevail [3,4]; (b) the dinuclear compound [Zn]_2_ is expected to have a key role in the transformation of the precursor into ZnO-based nanomaterials [14]; and (c) tiny changes in the aminoalcohol are important as to affect the N content in the final materials [4] and, therefore, their properties and potential applications. A deep knowledge of the number of steps and the changes involved therein would provide valuable information to minimize some of the problems of ZnO-based nanomaterials (e.g., presence of traces of N) and therefore to improve their quality for technological applications. However, the initial steps of the transformation of the precursor into ZnO and the role and relevance of the stabilizer still remain unknown. 

The computer power and the accuracy of theoretical approaches have recently increased significantly, allowing the use of computational tools to understand the behaviour of metal oxides such as ZnO. The utility of computational methods to understand the electronic and optical properties of ZnO has been reviewed [15,16] and their use is becoming more and more popular [17,18,19,20,21,22,23]. It is well known that Car-Parrinello molecular dynamics (CPMD) [24], combined with metadynamics algorithms [25], are powerful tools for studying a large variety of systems, from condensed-matter physics to chemical processes, and can provide a reasonable estimation of the successive paths of a chemical transformation [26,27,28,29,30,31,32,33].

In this paper, we present experimental studies based on infrared (IR), organic elemental analyses (OEA) and evolved gas analyses (EGA) of the evolution of the precursor towards ZnO, which provide relevant clues about the main changes occurring during the first steps of the process. Additional Car-Parrinello molecular dynamics (CPMD) combined with metadynamics simulations at different temperatures (300–770 K) have allowed the identification of the main species involved in the key steps of the process towards ZnO.

## 2. Experimental and Theoretical Methods

### 2.1. Materials and Procedures

The ZnO precursor was prepared by stabilizing 1.8 g of zinc acetate dihydrate (ZAD, from Panreac, Illinois Tool Works Inc., Castellar del Vallès, Spain) with an equimolar amount (0.47 mL) of ethanolamine (EA, from Acrös Organics, Thermo Fisher Scientific Inc., Geel, Belgium). The mixture was kept at 60 °C for 24 h under continuous stirring. Then, 7 mL of 2-methoxyethanol (ME, from Aldrich, Sigma-Aldrich Inc., St. Louis, MO, USA) was added as a solvent to prepare the ink, as reported previously [3]. All reagents were used as received. Afterwards, they were heated at 570, 670, 770 or 870 K at a rate of 10 K/min up to the desired temperature and were then quenched to room temperature without any delay to keep its structure after heating. 

The obtained samples were next characterized by infrared (IR) spectroscopy at room temperature with a Nicolet 400FTIR instrument (Thermo Fisher Scientific Inc., Waltham, MA, USA) using KBr pellets. Quantitative determinations of nitrogen, carbon and hydrogen content in the samples were carried out with a Thermo EA Flash 2000 (Thermo Scientific, Waltham, MA, USA) equipment working in standard conditions (helium flow of 140 mL/min; combustion furnace at 1223 K; chromatographic column oven at 338 K). Complementary Evolved Gas Analysis (EGA) of the evolved species was performed in a vacuum at a heating rate of 20 K/min using a quadrupole mass spectrometer (Microvision Plus, MKS Instruments. Spectra Products, Chesire, UK).

### 2.2. Quantum-Mechanical Calculations

Several approaches were used to describe the EA–ZAD compounds at the molecular level using Car-Parrinello molecular-dynamics [24] (CPMD package 2, version 3.15.1 [34]) simulations with Perdew-Burke-Ernzerhof, PBE [20], functional and ab initio pseudopotentials to describe the atomic potentials [35]. The plane-wave cut-off energy was set to 70 Ryd for the electronic wavefunctions (charge density). First, the geometries of the [Zn]_1_ and [Zn]_2_ complexes (shown in Figure 1) were optimized and their total energies were determined. After convergence of the wave functional and optimization, the external temperature was gradually modified. Afterwards, the evolution of chemical bonds with temperature was analysed by full CPMD calculations using a Nosé-Hoover thermostat [36] in the range between 300 and 770 K with a time step of 0.12 fs/step, applying a heating figure in which the temperature is held constant at values of 300, 570, 670 and 770 K for 600 integration steps. Finally, the main steps involved in the conversion of the dimer into ZnO were postulated thanks to the reconstruction of the free energy surfaces (FES) at 300 K by means of metadynamics calculations implemented in CPMD. After the identification of the most crucial species (transition states, intermediates and stable complexes), their structures were optimized at 0 K and their potential energy and potential energy surfaces (PES) were calculated.

The computational studies of [Zn]_1_ and [Zn]_2_ were carried out separately using a multistep strategy. This consisted of an initial test to determine the electronic kinetic energy (EKE, Table S1) for each one of the selected temperatures (from T = 300 K to 770 K). Simulation times up to 0.5 ps showed that a stationary state was reached before 0.25 ps, and, according to this, a simulation time of 0.25 ps was chosen as standard for these simulations. The EKE values obtained (Table S1) for the corresponding complex ([Zn]_1_ or [Zn]_2_) at a given temperature were used as input for the next step—the Nosé-Hoover [36] thermostat, with a frequency of 10,000 cm^−1^ for the electrons and 3500 cm^−1^ for nuclei, to run a 5000 MD step simulation. Afterwards, the temperature was slowly increased to the following T-value used in this study. This later step was repeated for each one of the remaining temperatures until the system reached 770 K.

## 3. Results and Discussion

### 3.1. Experimental Evidence

**Figure 2:**
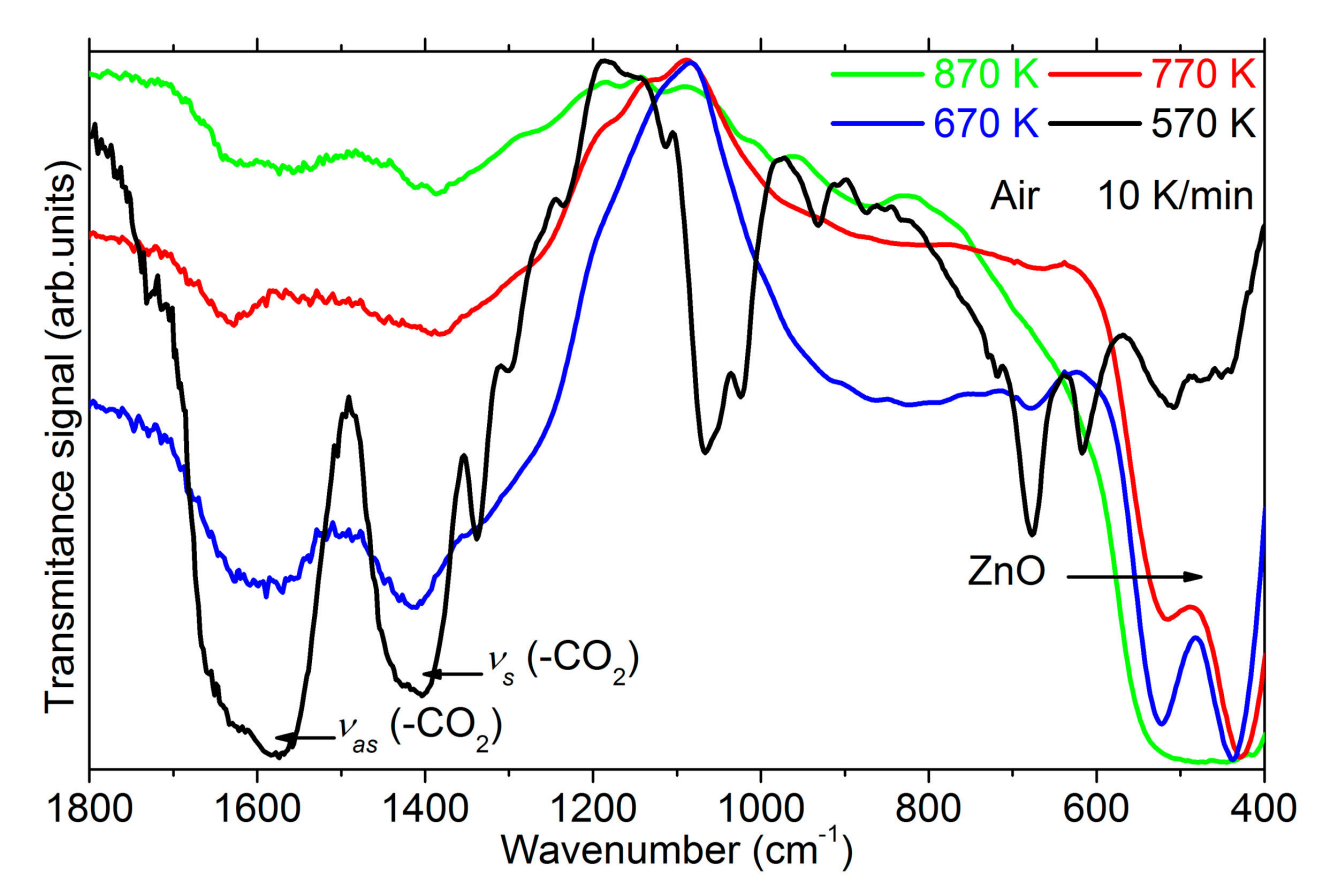
Infrared (IR) spectra of the precursor after heating up to 570, 670, 770 and 870 K at a rate of 10 K/min and immediate cooling down to room temperature.

In order to reach relevant information about the role of the stabilizer in the ink, some experimental analyses were undertaken. The structural evolution of the precursor was analysed by infrared (IR) spectroscopy and by elemental analysis. After heating at 570 K, the IR spectrum (Figure 2) shows the typical bands of (a) the asymmetric and symmetric stretching vibration modes of the bridging acetate ligands at ca. 1580 and 1400 cm^−1^ [37,38,39]; (b) the C–N and C–O groups due to the –NH_2_ and –OR moieties in the 1100–900 cm^−1^ range [40,41]; and (c) the wagging mode of –NH_2_ groups bound to transition metals around 680 cm^−1^ [40].

After heating at 670 K, the intensities of the bands due to the acetato ligands decrease, whereas those related to EA almost vanish. Moreover, the intense double band appearing below 600 cm^−1^ indicates the presence of ZnO [37]. These findings suggest that the initial step of the decomposition starts between 570 K and 670 K and it involves the cleavage of the Zn–N bond of the deprotonated EA. Elemental analysis of the sample after heating at 670 K confirms this hypothesis, revealing that the nitrogen content (1.3%) is significantly lower than in the fresh precursor (4.0%), whereas the C/N molar ratio increases from 4.0 in the precursor to 6.2 at 670 K.

At 770 K, the intensity of the bands due to organic ligands diminishes further, and at 870 K the typical Zn–O band reaches its maximum intensity. Besides, the elemental analysis shows that, even at the highest temperature tested (870 K), traces of organic residues still remain (0.6% C, <0.1% N and H). 

Complementary EGA experiments have given information about the nature of the volatile species leaving the precursor film as a function of temperature. Volatiles evolving from the sample, when heated, enter the ionization changer of the mass spectrometer where they are broken into fragments that are ultimately analysed by the spectrometer. A large number of fragments from *m/z* = 12 to 86 amu (atomic mass units) has been detected. When several volatiles evolve simultaneously, the intensity of some fragments results from the contribution of different volatiles rendering their identification difficult. To simplify the analysis, we have proceeded, as in our previous study on several aminoalcohols, [4] and considered that the fragments arising from a single volatile will follow the same temperature dependence. Consequently, the fragments have been classified according to their intensity dependence on temperature (details are given in Figure S1A–E) and one representative fragment of each group has been plotted in Figure S1F.

**Scheme 1:**
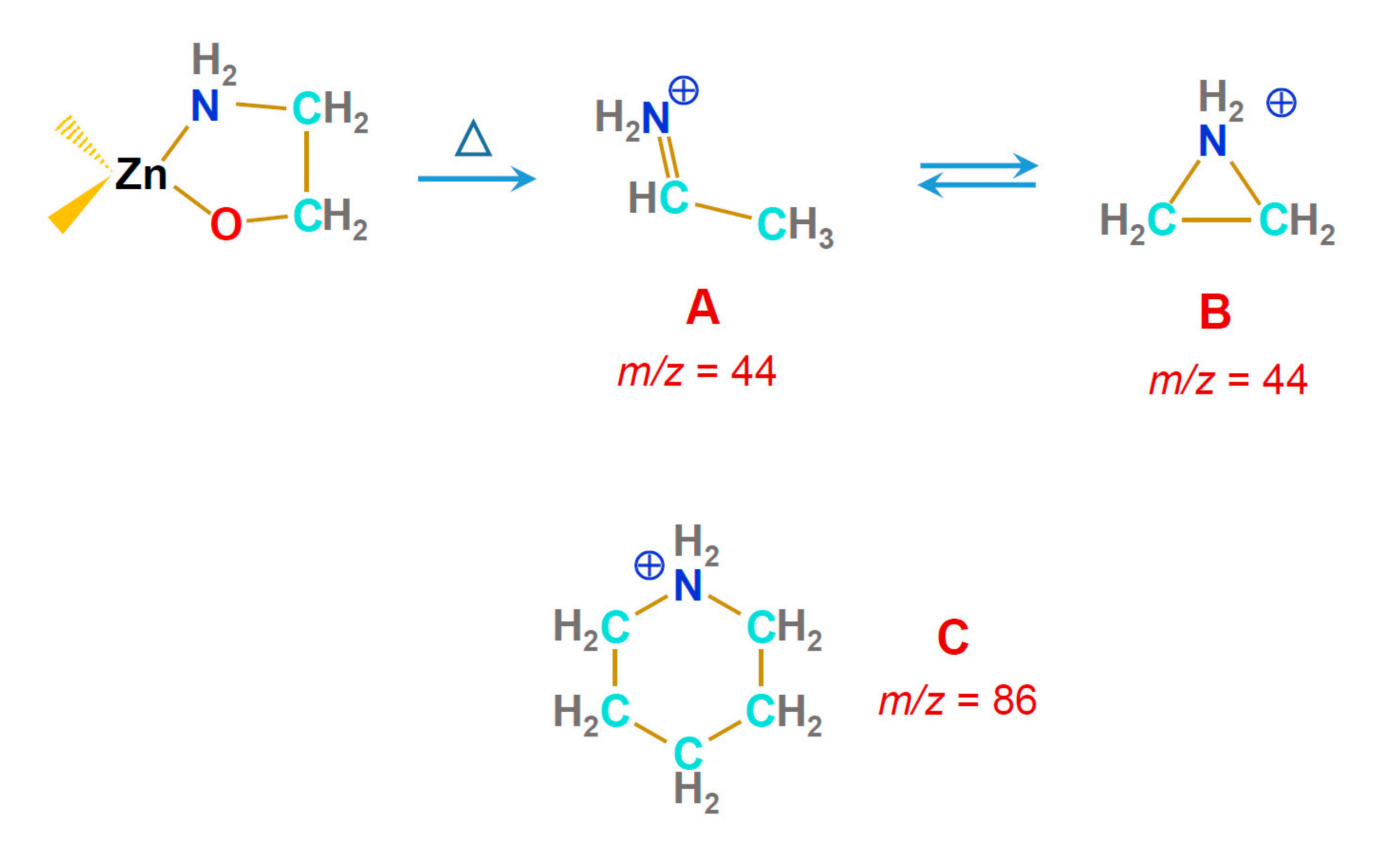
Simplified core of the precursor, in which the remaining coordination sites are occupied by acetate ligands, cation C_2_H_6_N^+^ in its linear (**A**) or closed (**B**) forms; and an example of one more complex N-containing cyclic cation (**C**) detected by EGA.

Apart from the cation with *m/z* = 58 amu, which may include other fragments besides the acetone formed during acetate decomposition [42,43], all the other peaks can be associated with a family of nitrogenated cyclic species identified by Bouchoux et al. [44] during EA dehydration. Among the observed sets, the one with highest intensity and detected in the widest temperature range is that related to *m/z* = 44 amu, C_2_H_6_N^+^, which could be produced after cleaving of the Zn–N bond in [Zn]_i_ (Scheme 1). According to Bouchoux [44], the initial linear configuration (A in Scheme 1) would evolve into the triangular cycle, which has a smaller energy (B in Scheme 1). All the other cyclic compounds (such as C in Scheme 1), whose *m/z* coincide with those of the EGA fragments of Figure S1, are the result of subsequent reactions beginning from the triangular cation [44].

### 3.2. Car-Parrinello Molecular-Dynamics Simulations

In order to get additional information on the effect produced by the temperature on the monomeric and dimeric Zn(II) model compounds (Figure 1), and especially on their Zn–(donor atoms) bonds’ lengths, full CPMD based on Density Functional Theory (DFT) [45] calculations for [Zn]_1_ and [Zn]_2_ complexes at different temperatures (300, 570, 670 and 770 K) were undertaken. As mentioned above, experimental data provide conclusive evidence that the formation of ZnO occurs at temperatures below 670 K. Therefore, the following discussion will be focused on the results obtained for T ≤ 670 K.

**Figure 3:**
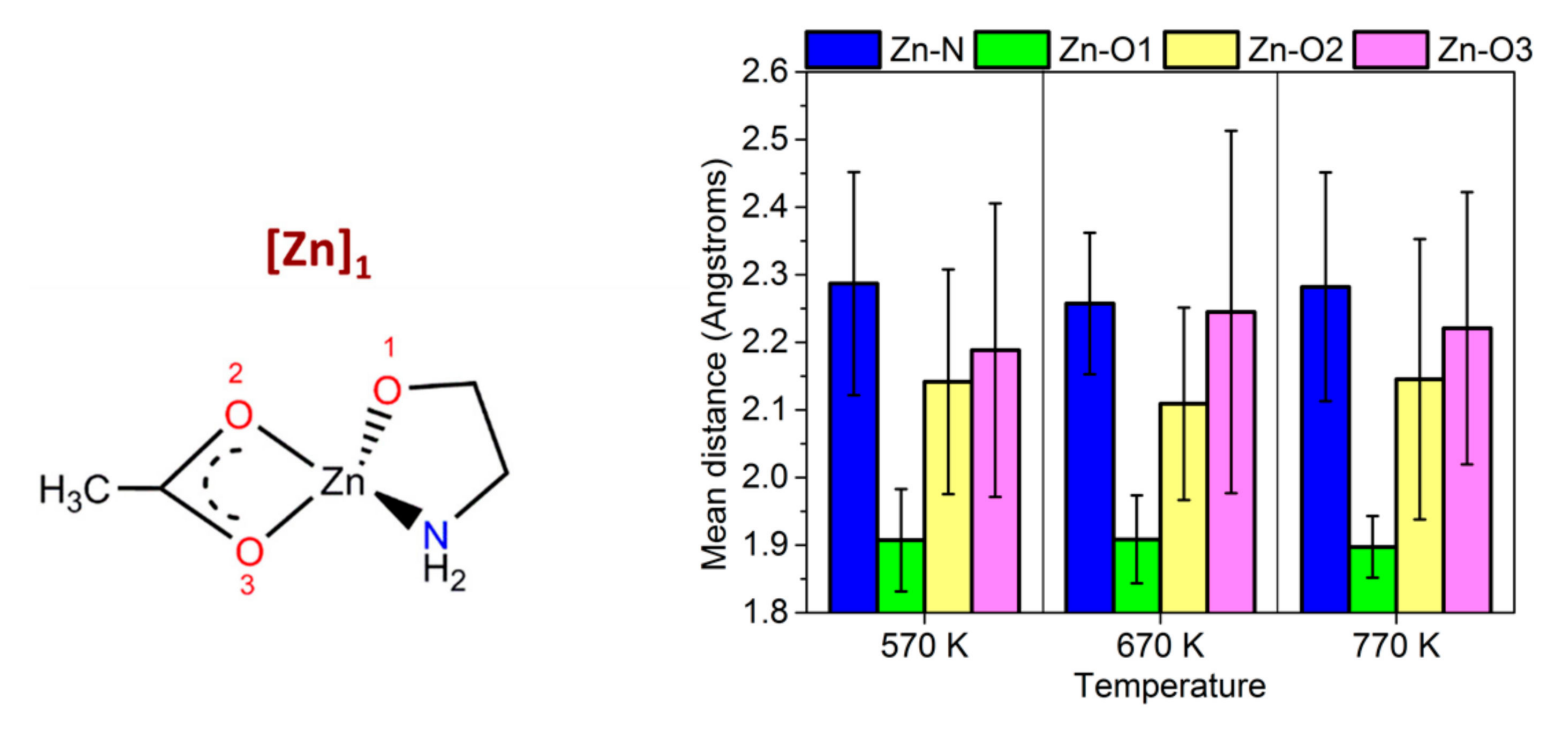
(**Left**) Chemical formulae of [Zn]_1_ showing the atom labels. (**Right**) Evolution of the mean Zn–N and Zn–O distances in [Zn]_1_ at 570, 670 and 770 K, using CPMD (a total of 2400 steps, 600 steps for each temperature). Vertical segments show standard deviations.

Figure S2 shows the evolution of the Zn–(donor atoms) bonds’ lengths in the monomer at T = 570, 670 and 770 K, and Figure 3 presents a summary of their mean values for [Zn]_1_ and their standard deviations (i.e., a quantification of the atomic oscillations).

The results obtained for the monomer provide valuable information on the changes produced by the temperature on the environment of the Zn(II) atoms and the binding modes of the ligands. First, comparison of the plots involving the Zn(II) and the two heteroatoms of the deprotonated EA (Figure 3) shows that there is not significant variation in the mean Zn–O1 bond length in the whole range of temperatures and this bond undergoes smaller oscillations (≈0.1 Å) than the Zn–N bond (≈0.4 Å). Moreover, in the three temperatures, the Zn–N bond distance clearly exceeds those reported for mononuclear Zn(II) complexes with H_2_N{CH_2_)_n_CH_3_} units bound to the Zn(II) atom [46,47]. In view of this, we postulate that during the first steps of the degradation process (which, according to IR studies, occurs between 570 and 670 K), the five-membered chelate ring “Zn(OCH_2_CH_2_NH_2_)” opens up and the binding mode of the deprotonated EA changes from (κ^2^-N,O)^–^ to (κ^1^-O)^–^. This generates a vacancy in the coordination sphere of the Zn(II) (we will return to this point later on) and a species with the “Zn–O(CH_2_)_2_NH_2_” unit that could undergo the cleavage of the Zn–O bond to release the cation C_2_H_6_N^+^ detected by EGA and shown in Scheme 1.

**Scheme 2:**
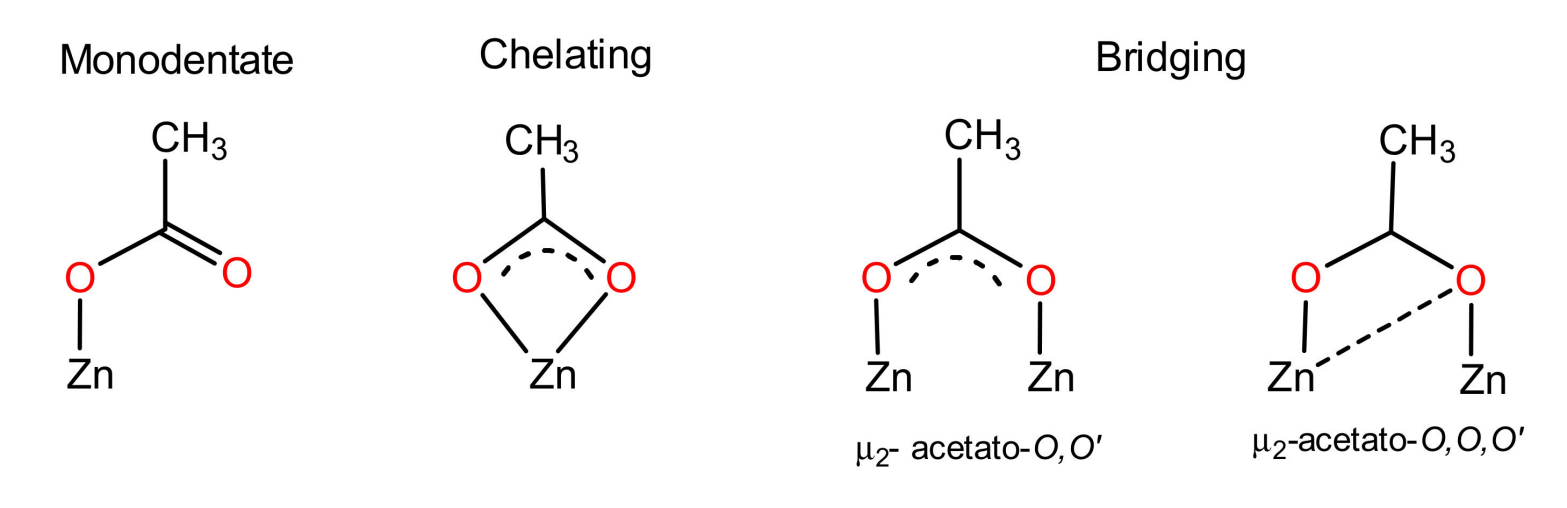
Schematic view of different binding model of one or two bridging acetato ligands.

The Zn–O(acetato) bonds’ (i.e., Zn–O2 and Zn–O3) distances are more “sensitive” to temperature changes than the Zn–O1. In fact, the differences observed in the plots (Figure 3) for the Zn–O2 and Zn–O3 bonds’ lengths suggest that the initial chelating acetato ligand present in the monomer also undergoes relevant changes. The variations observed for the mean values of the Zn–O3 bond length are larger than for the Zn–O2 bond. Consequently, the O3 atom tends to move further away from the metal centre than the O2. This is consistent with an asymmetric and chelating binding mode of the carboxilato ligands. At this stage, the resulting Zn(II) species formed after the cleavage of the Zn–N1 bond and the “reorganization” of the Zn–O(acetato) bonds may assemble. This would allow the filling of the vacant coordination position generated after the cleavage of the Zn–N1 bond, leading to compounds with higher nuclearity and bridging –OR ligands or acetato groups connecting two or three Zn(II) ions. Zn(II) complexes with μ_2_– and µ_3_–OR units are common [46,47,48,49,50,51,52,53,54,55,56,57,58,59]. They are present in a large amount of low-nuclearity compounds (i.e., dimers [46,48,49] or oligomers [46,50,51,52,53]), in the typical cubane-like “Zn_4_O_4_” clusters [46,49,54,55,56,57] and also in more sophisticated Zn(II) complexes [46,58,59] such as tetrakis {(µ_3_–methoxo)(pentacarbonyl-manganese)}zinc(II) [46,59]. Moreover, it is well-known that the acetato ligands may adopt a wide variety of coordination modes and denticities in Zn(II) compounds. Complexes of this type containing several acetato ligands acting as monodentate, chelating or as bridging (µ_2_–O,O’ or as µ_2_–O,O,O’) are known [46,60,61,62] (see Scheme 2).

**Figure 4:**
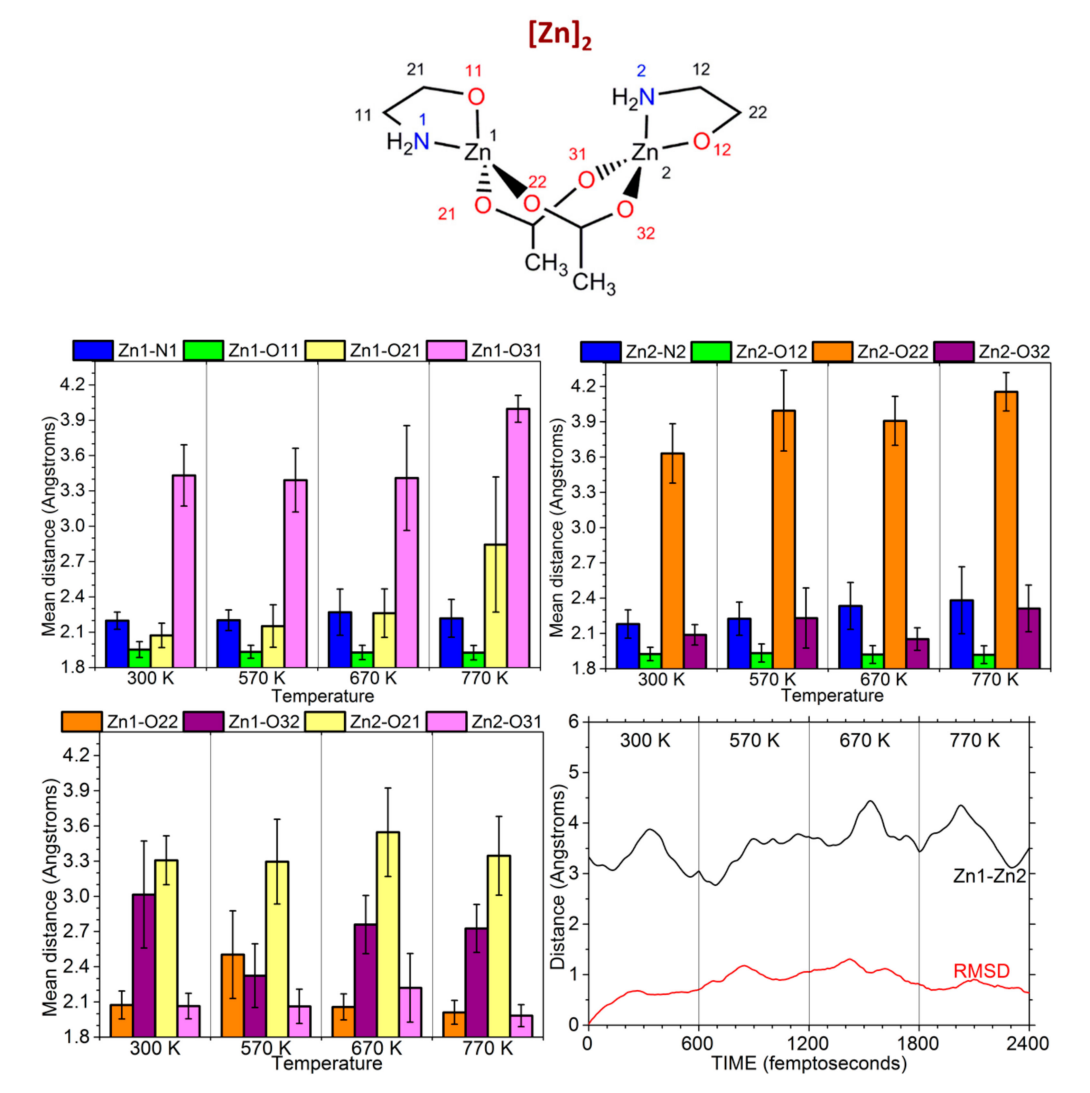
Chemical formulae of [Zn]_2_ showing the atom numbering scheme and evolution of the mean Zn–N, Zn–O and Zn···Zn distances and its root mean square deviation, RMSD, in [Zn]_2_ at 300, 570, 670 and 770 K (a total of 3000 steps, 600 steps for each temperature). Vertical segments show standard deviations.

Since the studies carried out with the [Zn]_1_ model suggested the formation of compounds of higher nuclearity after the opening up of the chelate and previous experimental studies revealed that degradation of the tetrameric precursor produced [Zn]_1_ and also [Zn]_2_ compounds, a parallel CPMD study with the dinuclear model was also undertaken. The results obtained (Figure 4 and Figure S3) show that there are no significant changes in the Zn1–O11 and Zn2–O21 bond distances for any of the temperatures under study; in contrast, the Zn1–N1 and Zn2–N2 bond distances, as well as their oscillation amplitudes, do increase with temperature. These trends are similar to those observed for the monomer [Zn]_1_ and indicate that, in the temperature range (570 K < T < 670 K, where the degradation starts), the opening of the five-membered chelate through cleavage of the Zn–N(EA) bond of the [Zn]_2_, is strongly preferred over that of the Zn–O(EA). This will lead to species containing the deprotonated EA acting as a monodentate (OR) ligand.

The central core of the [Zn]_2_ model also undergoes significant changes when the temperature increases from 300 K to 570 K. As shown in Figure 4, the distance between the two metal ions Zn1···Zn2 tends to increase and the Zn–O(acetato) bond lengths in each one of the two halves of the molecule, which were practically identical in the optimized geometry at 300 K, become different. This suggests a change in the electronic distribution around the Zn(II) atoms that involves the donor atoms of the acetato ligands. One of the acetato oxygen atoms gets further from its Zn atom (Figure 4 and Figure S3) and closer to the other one (e.g., Zn2–O32 bond enlarges from 2.09 Å at 300 K to 2.23 Å at 570 K, while Zn1–O32 distance diminishes from 3.01 Å at 300 K to 2.32 Å at 570 K). The proximity between this particular oxygen (O32) and the Zn1 atom enables the formation of a Zn1–O32 bond. As a consequence of this change, the central core “Zn1(µ_2_-acetato O,O’)_2_Zn2” loses its symmetry and turns into “Zn1(µ_2_-acetato–O,O’)(µ_2_-acetato–O,O,O’)Zn2.” This type of backbone is quite common in polynuclear derivatives [60,61,62]. The average values obtained from the computational studies for the Zn1–O32 bond length (2.329 Å), as well as for the Zn1···Zn2 distance (3.396 Å), are similar to those found in the literature for related derivatives holding simultaneously one (µ_2_-acetato–O,O’) and one (µ_2_-acetato–O,O,O’) units connecting two Zn(II) atoms (Zn–O bond lengths between 2.0 Å and 2.4 Å and the separation between Zn1 and Zn2 in the range 3.4–3.9 Å) [46,60,61,62].

In summary, the CPMD calculations for [Zn]_1_ and [Zn]_2_ models provide conclusive evidence that the first step of the degradation process involves the cleavage of the Zn–N(EA) bond. This is consistent with the results obtained from the IR spectra described above. The results also suggest that, for [Zn]_1_, the resulting species may assemble to fulfil the coordination sphere of the Zn(II) atom. In the dimer, the results point out that, after the cleavage of the Zn–N1 bond, there is a re-arrangement of the central core, which reduces the separation between the Zn1 and O32 atoms, allowing a µ_2_–O,O,O’ mode of binding of one of the acetato ligands.

Although the results obtained from the CPMD agree with the changes detected in the IR, they cannot yet completely explain two important points—(a) the formation of the triangular cation detected by EGA and (b) the presence of traces of nitrogen in the isolated ZnO samples even after treatments at high temperatures. It is widely accepted that CPMD suffers from a timescale problem, as its results are meaningful only if the run visits all the energetically relevant configurations. In the case that high free-energy barriers separate metastable states, the change from one to another can take place only if activated by those rare fluctuations that can take the system over the barrier. Under these conditions, obtaining sufficient statistics requires an impractical amount of computer time. In these cases, a suitable methodology for accelerating events (reactions, bond cleavage, etc.) is needed. Among the methods frequently used to scan all the configurations and deepen in the process comprehension, Metadynamics simulations are by far the preferred ones.

### 3.3. Metadynamics Simulations

In order to get further information on the changes involved during the first steps of the [Zn]_2_ decomposition, as well as to understand the variations detected in the IR spectra and the results obtained from EGA, additional metadynamics simulations were carried out. As mentioned above, the formation of the triangular cation with *m/z* = 44 should arise from the deprotonated and bidentate EA units of [Zn]_2_. This requires the cleavage of the Zn–N and O–C(EA) bonds of at least one of the two units present in the dimer. With this idea in mind, a metadynamics calculation was performed using the CPMD software. In this study, the geometry of the dimer obtained from the molecular dynamics simulation was used as the input and was subject to the activation of the Zn–N and O–C bonds of one of the two deprotonated EA, in order to reconstruct the FES. Due to the symmetric nature of the [Zn]_2_, in a first stage we arbitrarily selected the Zn1–N1 and O11–C21 bonds. After the optimization, the [Zn]_2_ simulation was restarted from the same temperature; during a 9.4 ps MD simulation (0.12 fs per step), 391 repulsive Gaussians (deposition time of 200 MD steps) have been applied to allow the system to evolve over the FES. The height of the Gaussian was 1 kcal/mol and the width σ was 0.15 a.u. (for the Zn1–N1 bond) and 0.075 a.u. (for the O11–C21 bond).

**Figure 5:**
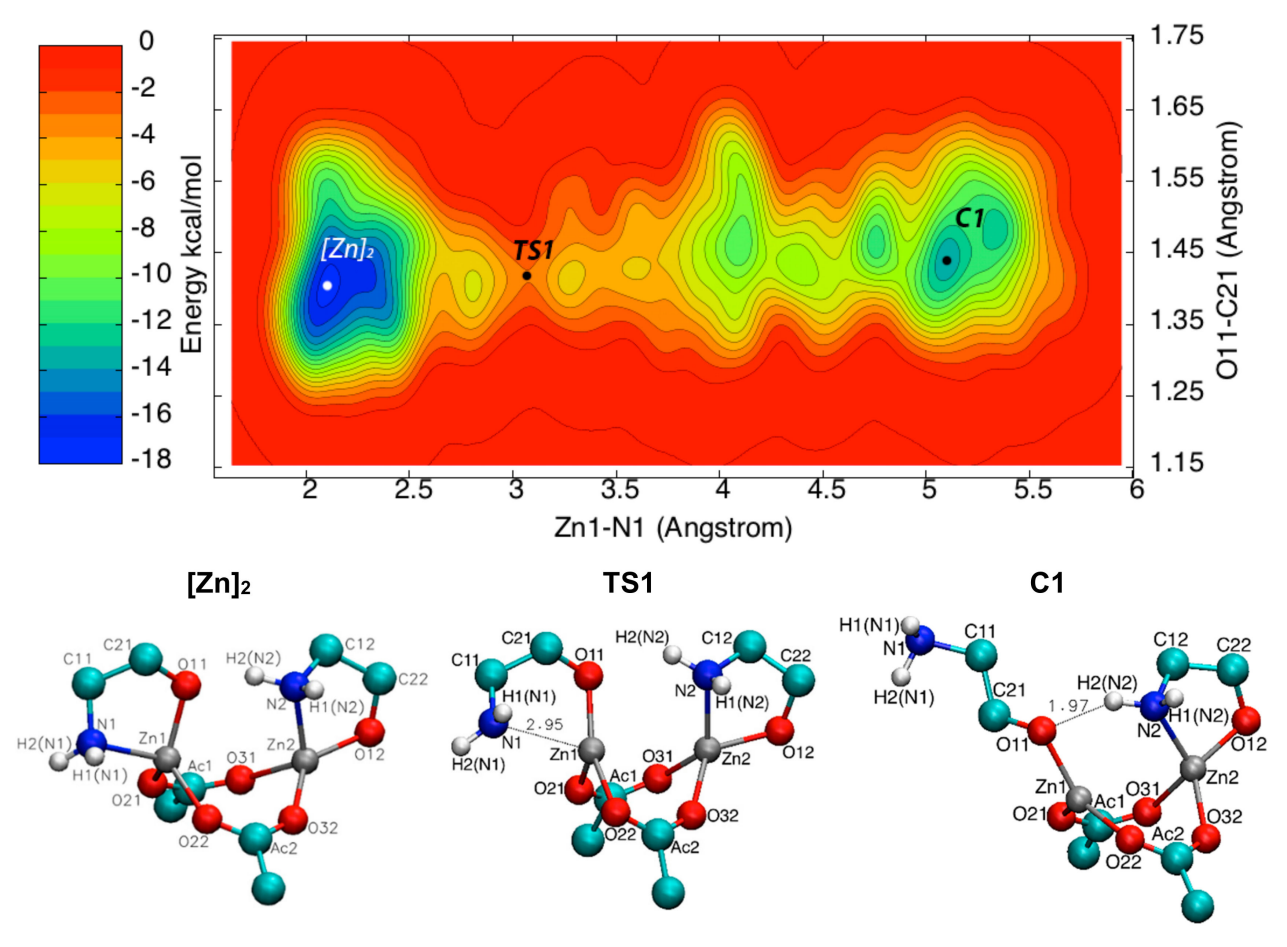
Labelling schemes and free energy surface (FES), as calculated by metadynamics. **C1** corresponds to an energy minimum, while TS1 is a transition state.

The process leading to Zn1–N1 bond cleavage can be understood thanks to the dimer metadynamics, the results of which are shown in Figure 5, in good agreement with the values reported for dimeric compounds with similar cores [46,63]. Despite the fact that many transient structures may exist for times smaller than femtoseconds, from now on we will focus exclusively on the main species (i.e., transition states and intermediates) and the chemical changes involved in the process under study.

**Scheme 3:**
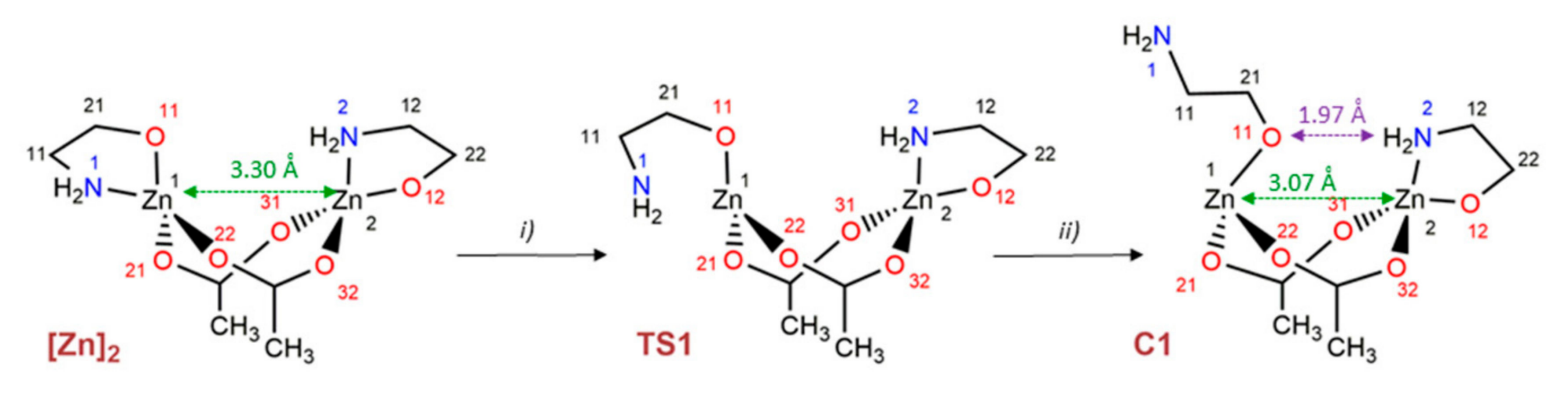
Sequence of initial steps involved in the dimer modification before the formation of the triangular cation detected by EGA.

The final atomic coordinates for the optimized structures of [Zn]_2_ and **C1** together with the electrostatic potential (ESP) charges of all atoms are summarized in Tables S2 and S3, respectively. Simplified views of the conformational or structural modifications occurring during the conversion of [Zn]_2_ into **C1** are shown in Scheme 3. The corresponding variation in interatomic distances obtained from [Zn]_2_ to complex **C1** is presented in Figure S4, which clearly shows that the biggest distance fluctuations are observed for Zn1–N1, as well as between O11 and H2(N2), reaching values above 3.5 Å.

The process [Zn]_2_ → **C1** may be described as—(a) cleavage of Zn1–N1 bond and consequent ring opening; (b) reorientation of the pendant “CH_2_–CH_2_–NH_2_” unit bonded to O11; (c) approach of O11 to the right half, allowing an intramolecular O11···H2–N2 contact and, as a consequence of this, decreasing of the Zn1···Zn2 distance from 3.30 to 3.07 Å. Besides, the comparison of data in Tables S2 and S3 reveals significant variations of the charges of the main atoms participating in the process. All these findings are consistent with the CPMD results for the [Zn]_2_ species described above, which suggested the prevalence of the Zn–O bond over the Zn–N bond, and pointed out that the first step of the decomposition process involves a change of denticity and binding mode of the coordinated EA from bidentate (κ^2^-N,O)^−^ to monodentate (κ^1^-O)^−^.

Figure S5 presents the variations of the free and potential energies (FES and PES, respectively) in relation to those of the dimer under identical conditions. As shown by the PES curve, at 0 K, the energy of **C1** and the barrier attributed to **TS1** are rather high (18 and 20 kcal/mol, respectively), indicating a negligible probability of evolution from [Zn]_2_ to **C1** at 0 K. However, at 300 K, the calculated energy for **C1** (−13.8398 kcal/mol) is only ca. 3.6 kcal/mol higher than that of the dimer (−17.4072 kcal/mol). The energy barrier decreases (from 20 kcal/mol at 0 K to 14 kcal/mol at 300 K) and it is expected to be more and more accessible at higher temperatures, for example, when the decomposition starts (570 K < T < 670 K).

As shown in Figure 5 and Scheme 3, **C1** contains one EA bound to the Zn1 through the oxygen O11. The results obtained from this metadynamics simulation revealed that the C21–O11 bond could not be broken, even adding 60–70 kcal/mol. Consequently, the formation of the triangular cation from **C1** is not likely to occur and should arise from different species formed in the subsequent steps of the process. It should be noted that, during the first steps of this simulation, the structure of the complex molecule changed its shape, giving a new species the structure of which was optimized, and the electrostatic potential (ESP) charges of its atoms were calculated. Afterwards, CPMD at 300 K of this intermediate state was performed over 1.8 ps leading to a new structural change. Free rotation of the pendant EA arm may allow a proper orientation between the N1 atom and Zn1 as to bind and therefore regenerate the dimer.

Besides, these MD simulations revealed that the O11 tends to approach the H2(N2) and Zn2 atoms [distances O11··· H2(N2) and O11···Zn2 decreasing from 1.97 Å and 4.0 Å (in **C1**) to 1.79 and 3.8 Å respectively] to produce a new compound **C2** (Figure S5 and Table S4), in which one of the acetato groups acts as a µ_2_-O,O,O’ bridging ligand. This arrangement, also found in other Zn(II) complexes [46,64,65], agrees with the conclusions reached from the analyses of the variations detected in the Zn–donor atoms’ bond obtained by CPMD and described above. 

In the next step, a 1.8 ps MD simulation of **C2** at 300 K showed that the system evolves to a new product **C3**. Its optimized geometry (Figure S5 and Table S5) shows that: a) the Zn1···Zn2 distance decreases to 3.0 Å; b) the oxygen O11 of the pendant arm in **C2** now bridges both zinc atoms, and c) the C21–O11 bond elongates in relation to that of **C1**. In fact, in **C3,** this bond distance (1.45 Å) is clearly larger than those found in Zn(II) compounds with one bridging O–CH_2_–CH_2_–NR_2_ ligand (i.e., R = Et or Me) [46,66,67]. Since the C21-O11 bond in **C3** is weaker than in **C1**, its cleavage is more prone to occur releasing the C_2_H_6_N^+^ array that later on transforms into the triangular cation with *m/z* = 44 detected by EGA.

In compound **C3,** the Zn2 atom still remains bound to a bidentate EA ligand, which could also undergo a ring opening process similar to that involved in the transformation [Zn]_2_→ **C1**. In view of this, and as a first approach to this problem, a 1-Collective Variable (CV) metadynamics simulation with the activation of the Zn2–N2 bond was next undertaken. After a 0.6 ps molecular dynamics at 300 K, 126 repulsive Gaussian potentials (deposition time 200 MD steps) were applied over the mono-dimensional FES along a 3.07 ps MD. The height of the Gaussian was 1 kcal/mol during the first ps, and it was decreased to 0.5 kcal/mol during the rest of the process. The width σ was 0.20 a.u. Finally, a representative structure of the products was optimized and its ESP charges were calculated.

As shown in the 1D free energy profile (Figure S6), several energy minima appear in the range of Zn2-N2 distances between 2.5 and 5.5 Å, of which the first value corresponds to **C3**. The energy barriers of the initial transformations [∆E_1_, ∆E_2_ and even ∆E_3_] are small (0.6, 0.7 and 1.9 kcal/mol, respectively). The last step with a ∆E_4_ smaller than that of the [Zn]_2_ →**C1** (Figure S5) leads to compound **C4**, which results from the cleavage of the Zn2–N2 bond and the subsequent change in the acetato binding mode. Bond lengths and angles of the optimized geometry of **C4** (see Table S6 for final atomic coordinates) are consistent with the values found in Zn-complexes with bridging acetato and –OR ligands [46,68]. In **C4**, the C21–O11 bond length (1.46 Å) is quite similar to that of **C3** (1.45 Å) and larger than in **C1.** This suggest that, in **C3** and **C4**, the release of the C_2_H_6_N^+^ cation through the cleavage of is more likely to occur than in **C1**. 

It is well-known that mono-, di-, tri- or even polymeric Zn(II) complexes with central diamond-like “Zn(µ_2_–OR)_2_Zn” units are relevant in coordination chemistry and in materials sciences. Compound **C4** has one “Zn(µ-OCH_2_CH_2_NH_2_)Zn” and an additional monodentate EA bond that could also undergo a change in its binding mode similar to that occurring in the first steps of the process [Zn]_2_→**C1**→**C2**. This would lead to species with “Zn(µ_2_–OR)_2_Zn” units, of which the simplest example would be the dimeric product **P** with two bridging deprotonated EAs. 

In view of this, we also tested the viability of the transformation of **C4** into a dimeric compound (**P**) with two bridging deprotonated EA ligands. It should be noted that theoretical description of the path bringing from **C4** to **P** at 300 K involves metadynamics with too many collective variables to be activated and analysed. Therefore, the FES of the transformation of **C4** into **P** cannot be accurately achieved. Consequently, as a first approach to clarify whether the existence of the diamond-like core could induce significant variations in the strengths of the O–C EA bond, the optimization of the geometry of **P** was carried out. The final atomic coordinates are presented in Table S7 and the bond lengths and angles are consistent with those reported for related compounds with diamond-like “Zn(µ–OR)_2_Zn” units [46,69,70]. In the optimized geometry of **P**, the O11–C21 and O12–C22 bonds are longer than those of **C1** and quite similar to those of **C2**, **C3** and **C4**.

**Scheme 4:**
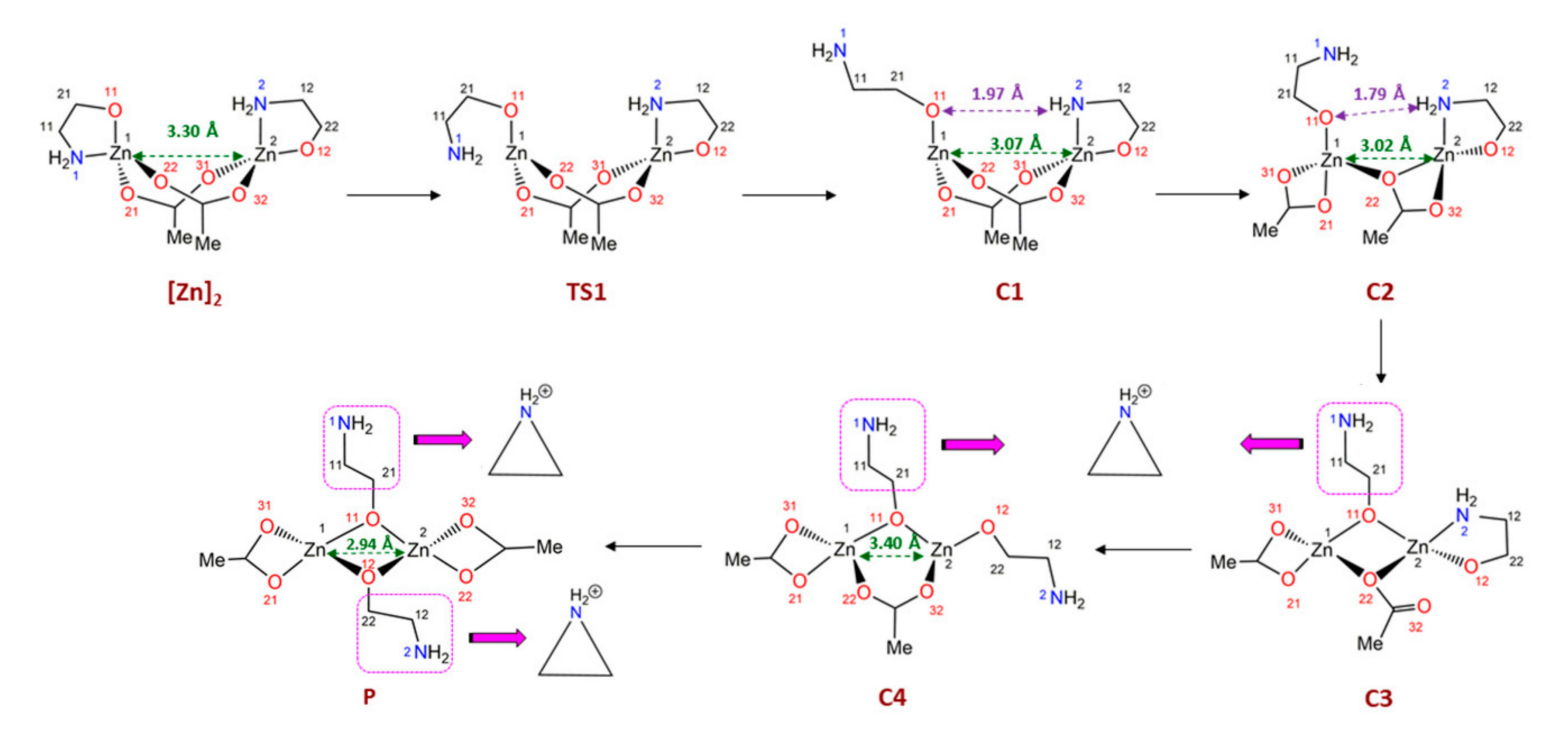
Complete dimer modification to generate a triangular molecule, according to the obtained results.

As the main conclusion of this analysis, a decomposition path is proposed in Scheme 4. It is coherent with all the descriptions made until here, but also with detailed descriptions of the evolution of the ZAD plus EA precursor during decomposition [3].

Moreover, the elongation of the C–O bonds from **C1** (Tables S2–S7) suggest that the release of the triangular cation C_2_H_6_N^+^ from **C1** is less likely to occur than in **C2-C3-C4** and in the model complex **P** that has a diamond-like core. The decomposition mechanism proposed in this paper allows the molecule to readjust itself to weaken one among the C–O bonds and form the triangular species, in good agreement with the experimental results presented above. Although the calculation approach summarized here suggests that the formation of the triangular cation may arise from any of the **C2**, **C3** or **C4**, the cleavage of the C–O bond of EA may occur at different rates and could explain the presence of N traces in the final ZnO product.

Consequently, tiny changes in the backbones of aminoalcohols derived from EA by the replacement of one or more of the H atoms of the CH_2_ units by other substituents may be important as to modify the charges of the heteroatoms and/or induce significant steric effects, which could affect the stability of species and transition states formed in the initial steps of the thermal decomposition, as observed for closely related precursors with amino-propanol and amino-methyl-butanol [4]. 

## 4. Conclusions

The studies summarized in this work provide conclusive evidence of the relevance of the ethanolamine used as a stabilizer in the sol-gel formation of ZnO. Experimental studies point out that the first step of the thermal conversion of the precursor formed in the reaction of ZAD with EA in ME into the ZnO involves cleavage of the Zn–N bond and the opening of the five-membered chelate ring. 

The results obtained from CPMD studies included in this work indicate that the Zn–O(EA) bond length does not change significantly in the range of temperatures selected (up to 770 K), while the Zn–N bond elongates. Moreover, the variations detected in the Zn–O(acetate) bond lengths in the dimer, as well as the distances between the two metal ions, suggest that during decomposition the central “[Zn(µ-AcO)_2_Zn]“ core undergoes significant structural modifications (change of the binding mode and hapticity of one of the AcO^−^ ligands). All these findings are consistent with the variations detected in the IR spectra.

Additional metadynamics simulations provide an overview of the dimer evolution by the cleavage of the Zn–N bond and shows the relevance of (i) the hydrogen atoms attached to the amino group; and (ii) the intermolecular N–H···O interactions in intermediate species, which play a key role in the initial steps of the process. Consequently, tiny changes in the backbones of aminoalcohols derived from EA by the replacement of one or more of the H atoms of the CH_2_ units by other substituents may be important as to (a) modify the charges of the heteroatoms; (b) induce significant steric effects or (c) both simultaneously, which could affect the stability of species and transition states formed in the initial steps of the thermal decomposition. Furthermore, they demonstrate that the formation of “Zn(µ-OCH_2_CH_2_NH_2_)Zn” cores affects the strength of the C–O EA bond, making its cleavage easier and, therefore also the release of the “C_2_H_6_N^+^“ cation (linear or triangular).

## Supplementary Materials

The following are available online at https://www.mdpi.com/2079-4991/9/10/1415/s1, Figure S1: Normalized EGA signals with their required normalization factor in parenthesis classified into five groups (**A–E**) according to their mean *m/z* values. In **F**, representatives of every group with their required normalization factor in parenthesis, Figure S2: Evolution of the Zn–N and Zn–O distances in [Zn]_1_ at 570, 670, 770 and 870 K, using CPMD, Figure S3: Evolution of the Zn–N and Zn–O distances in [Zn]_2_ at 300, 570, 670, 770 and 870 K, Figure S4: Variation in interatomic distances, as given by the first metadynamics simulation, from [Zn]_2_ to complex **C1**, Figure S5: Free and potential energy profiles of the decomposition process, and optimized geometries of the transition state **TS1** and complexes **C1-C3**, Figure S6: 1D free-energy profile plotted in terms of the Zn2–N2 distance, Figure Figure S7: Free and potential energy profiles for the transformation C3 → C4, and calculated energy for complex P, Table S1: Electronic Kinetic Energies obtained for the systems [Zn]_1_ and [Zn]_2_ at different temperatures, Table S2: Final atomic coordinates for the optimized geometry of [Zn]_2_ and calculated charges, Table S3: Final atomic coordinates for the optimized geometry of complex **C1** and calculated charges, Table S4: Final atomic coordinates for the optimized geometry of complex **C2** and calculated charges, Table S5: Final atomic coordinates for the optimized geometry of complex **C3** and calculated charges, Table S6: Final atomic coordinates for the optimized geometry of complex **C4** and calculated charges, Table S7: Final atomic coordinates for the optimized geometry of the final **P** complex and calculated charges.
