# Supplementary material for: From Ethanolamine Precursor Towards ZnO—How N Is Released from the Experimental and Theoretical Points of View
Source: Nanomaterials (Basel). 2019 Oct 3;9(10):1415. doi: 10.3390/nano9101415 (PMC6835746; doi:10.3390/nano9101415)
Supplement: Supplementary file 1 [file nanomaterials-09-01415-s001.pdf]

Supplementary information

From Ethanolamine Precursor Towards ZnO—How N is Released from the Experimental and Theoretical Points of View

Alberto Gómez-Núñez [1,2,3], Santiago Alonso-Gil [4,5], Concepción López [4], Pere Roura-Grabulosa [6] and Anna Vilà [1,2,*]

1 Department of Electronic and Biomedical Engineering, University of Barcelona, Martí i Franquès 1, 08028 Barcelona, Spain
2 Institute of Nanoscience and Nanotechnology (IN2UB), University of Barcelona, Joan XXIII s/n, 08028 Barcelona, Spain
3 FAE Francisco Albero S.A.U., Rafael Barradas 19, Granvia L'Hospitalet Economic District, 08908 L'Hospitalet de Llobregat, Spain; agomez1618@gmail.com (A.G.-N.)
4 Department of Inorganic and Organic Chemistry, University of Barcelona, Martí i Franquès 1, 08028 Barcelona, Spain; conchi.lopez@qi.ub.es (C.L.)
5 J. Heyrovský Institute of Physical Chemistry, Czech Academy of Sciences, Dolejškova 2155/3, 18223 Prague 8, Czech Republic; santi_galdor_quantum@hotmail.com (S.A.-G.)
6 Department of Physics, Campus Montilivi, University of Girona, Edif. PII, 17003 Girona, Spain; pere.roura@udg.cat (P.R.-G.)
* Correspondence: anna.vila@ub.edu; Tel.: +34-93-4039170

Received: 4 September 2019; Accepted: 29 September 2019; Published: date

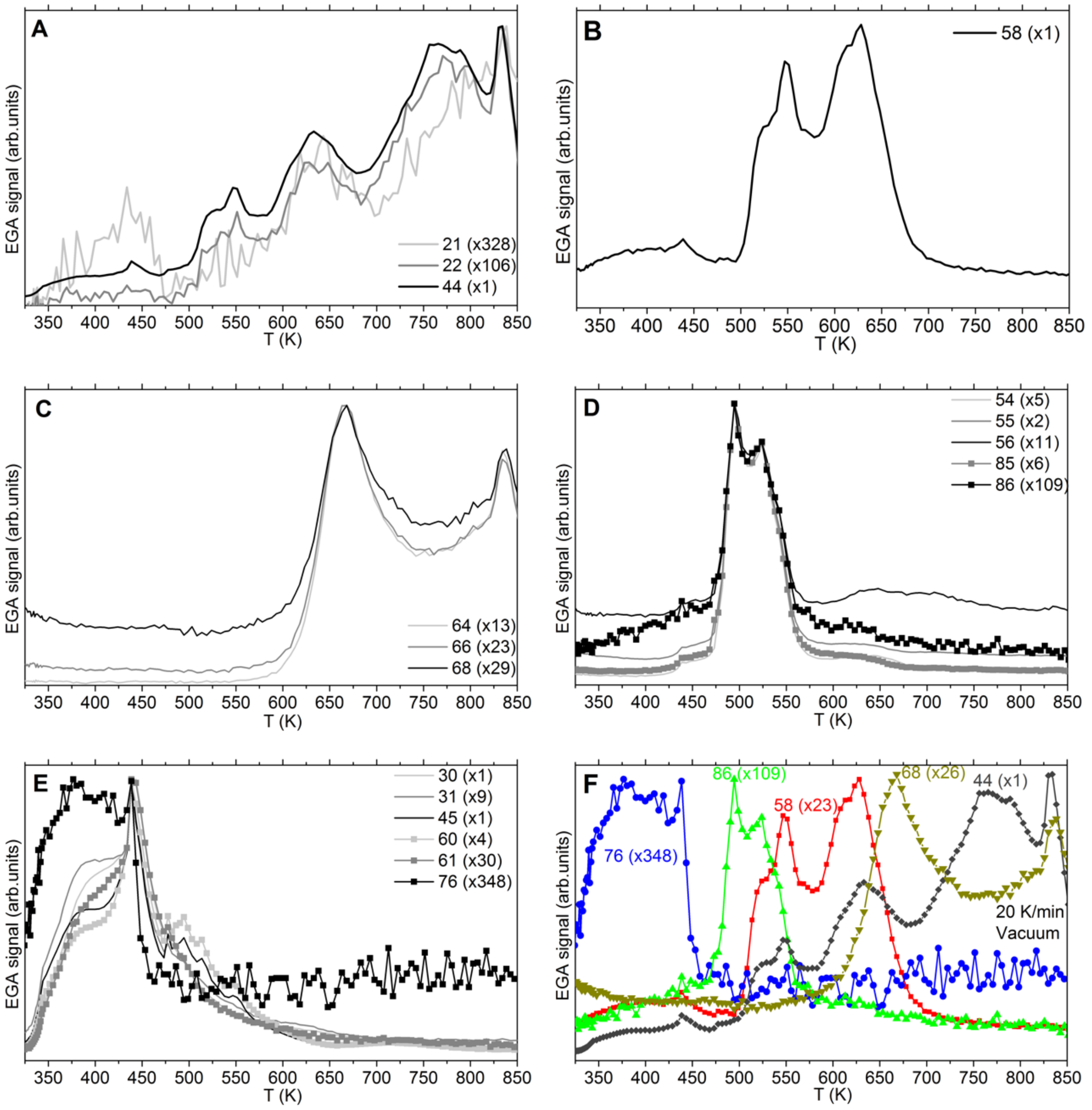

Figure S1. Normalized EGA signals with their required normalization factor in parenthesis classified into 5 groups (**A**–**E**) according to their m/z values. In **F**, representatives of every group with their required normalization factor in parenthesis.

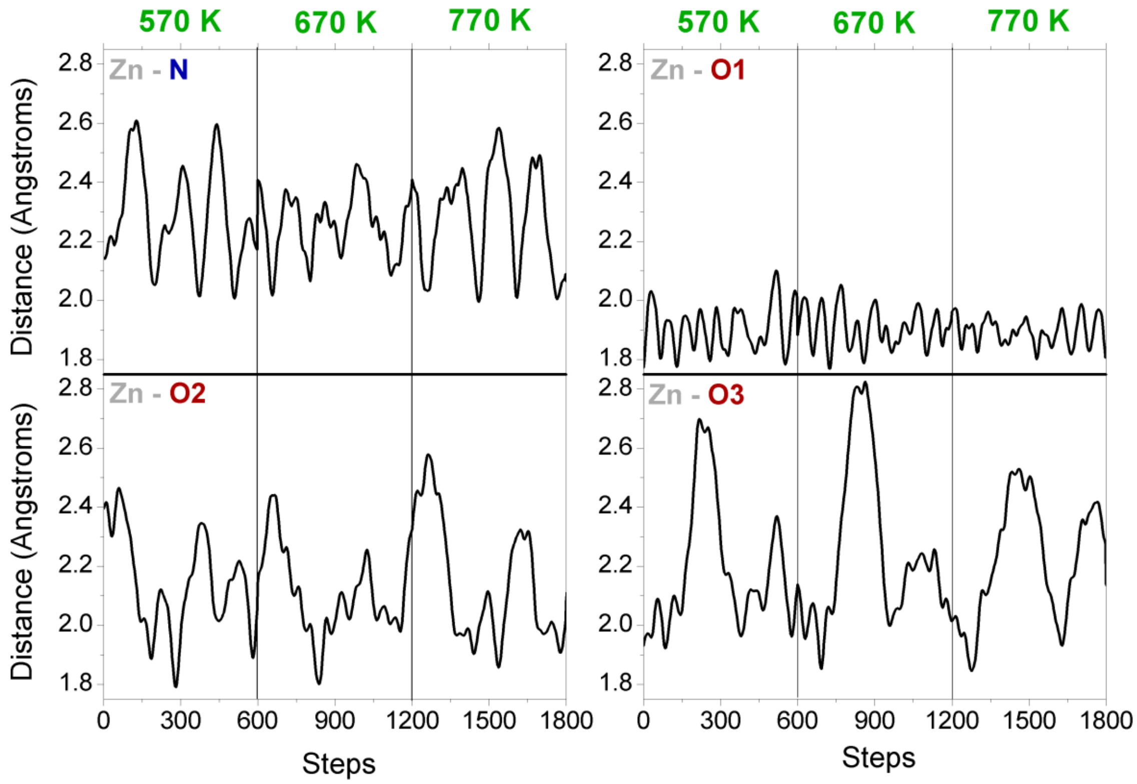

Figure S2. Evolution of the Zn–N and Zn–O distances in $[Zn]_1$ at 570, 670 and 770 K, using CPMD.

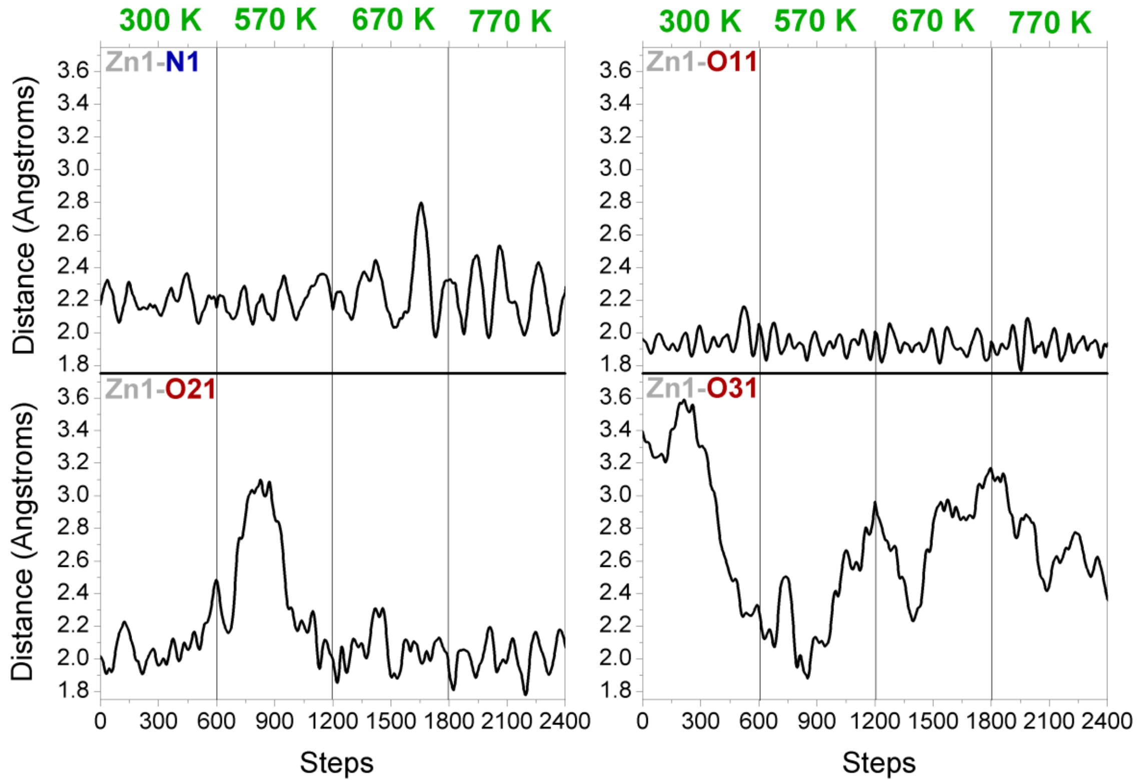

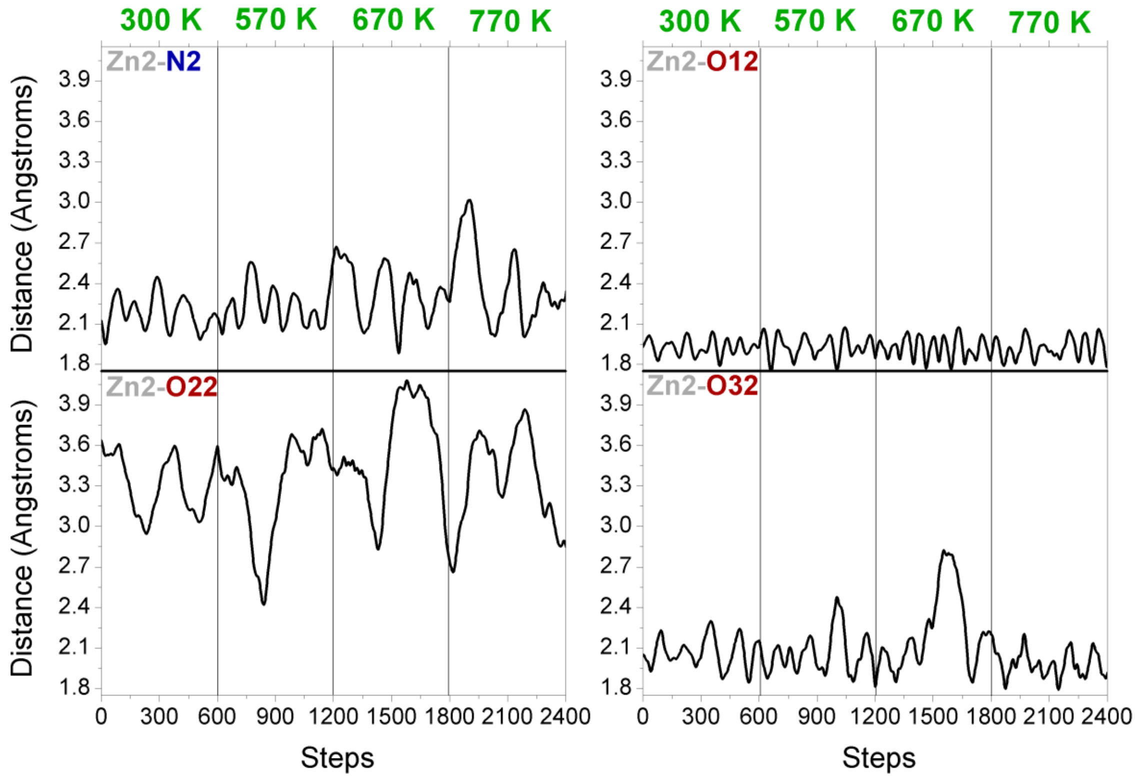

Figure S3. Evolution of the Zn–N and Zn–O distances in $[Zn]_2$ at 300, 570, 670, 770 K.

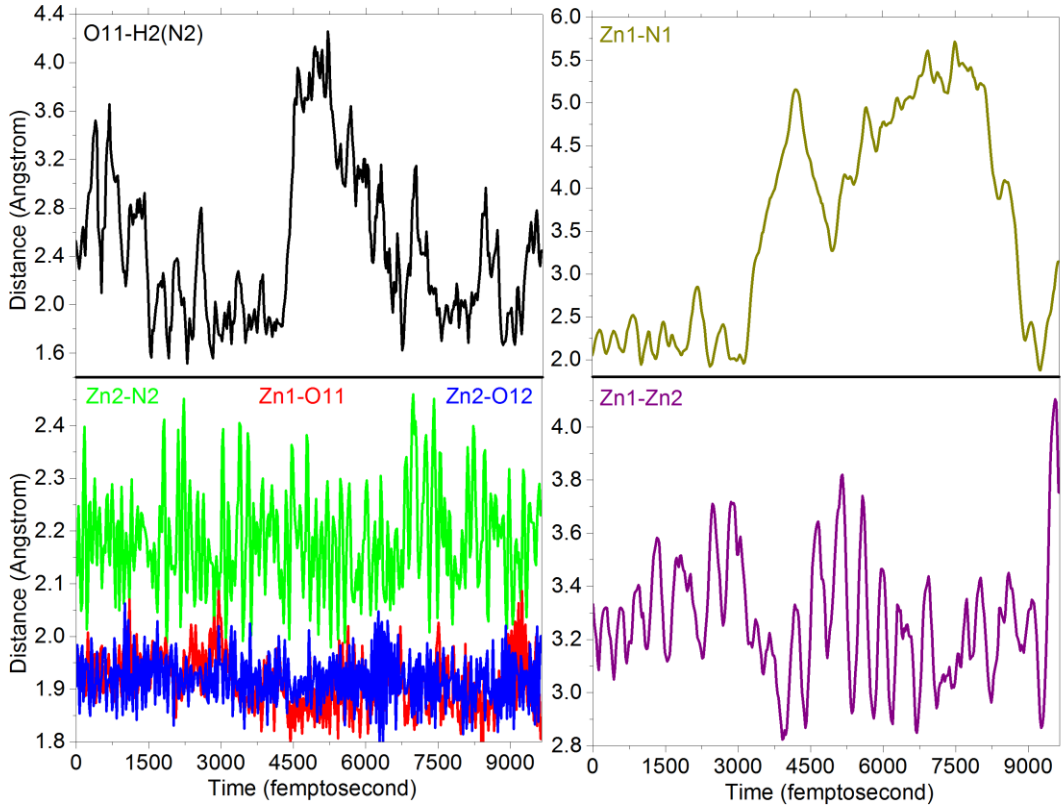

Figure S4. Variation in interatomic distances, as given by the first metadynamics simulation, from $[Zn]_2$ to complex **C1**.

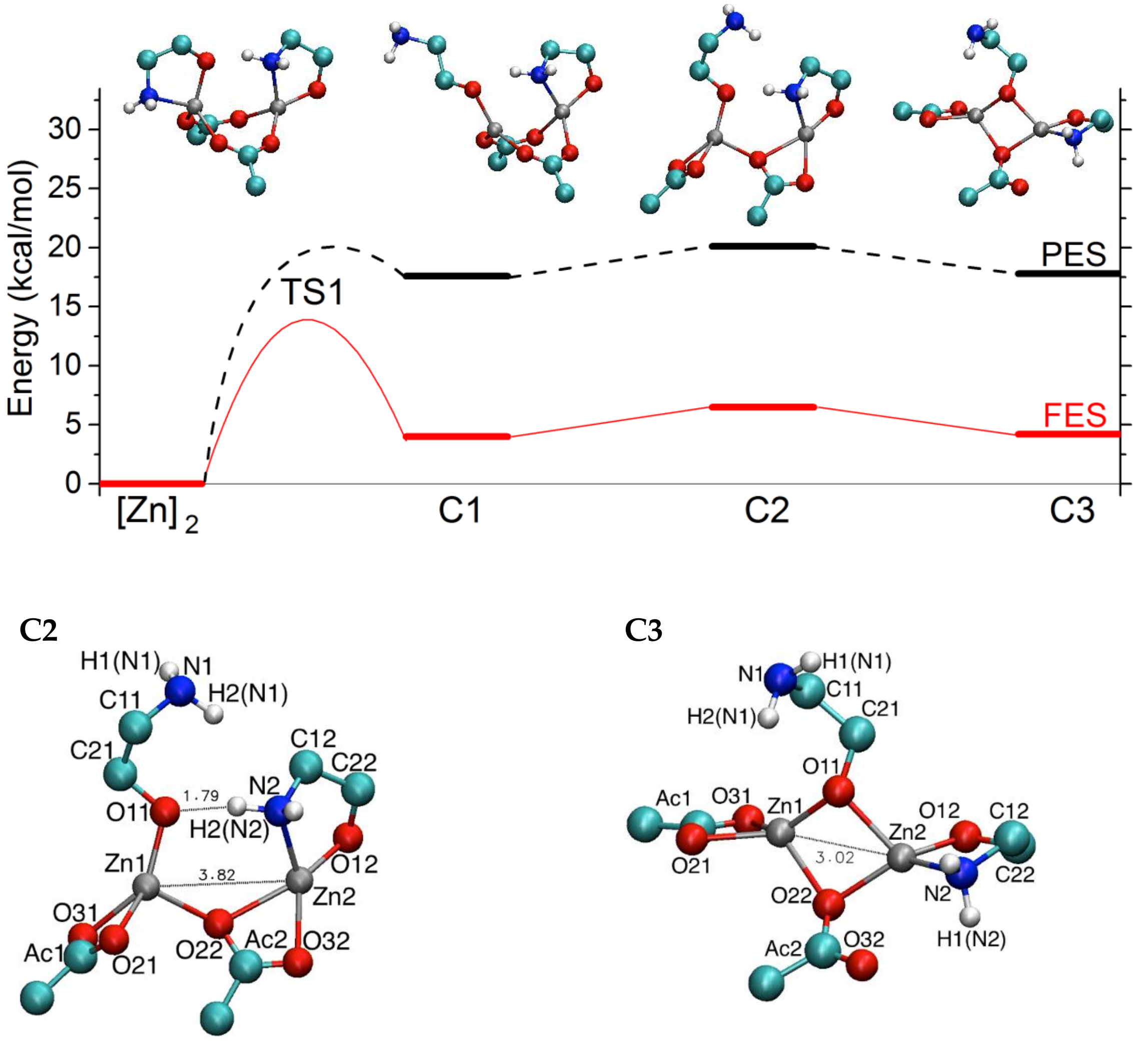

Figure S5. Free and potential energy profiles of the decomposition process, and optimized geometries of the transition state **TS1** and complexes **C1-C3**.

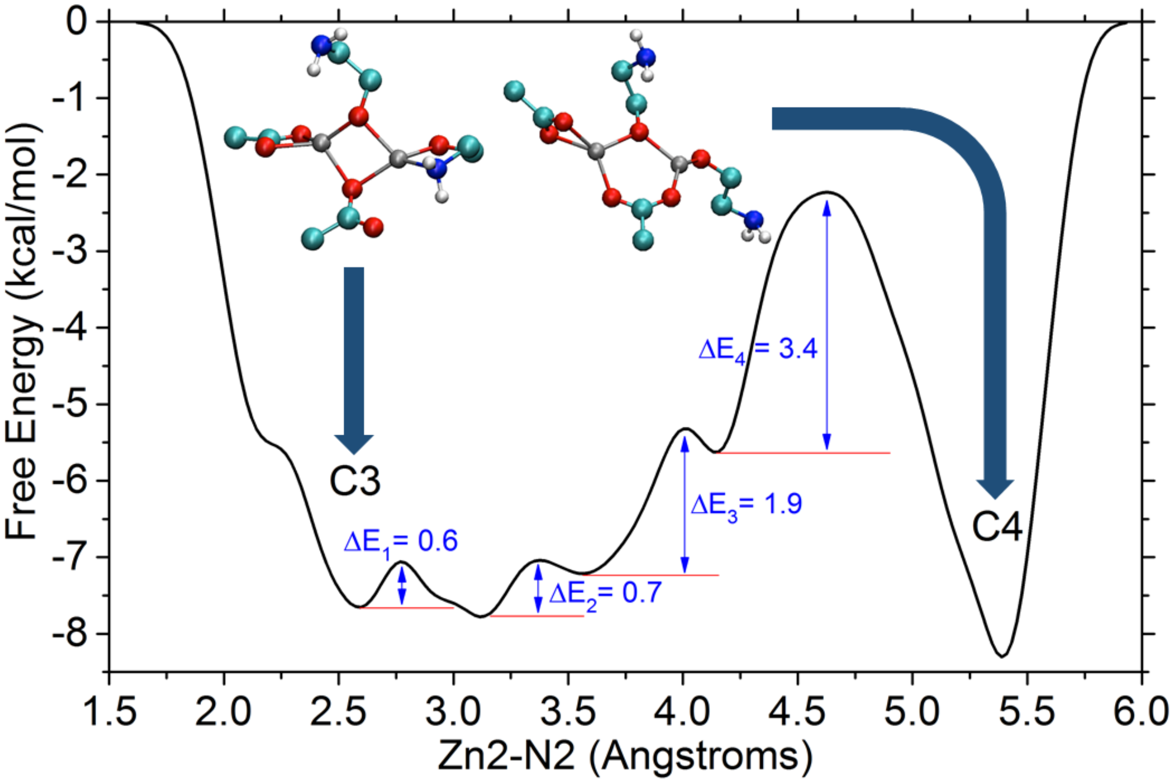

Figure S6. 1D free-energy profile plotted in terms of the Zn2–N2 distance.

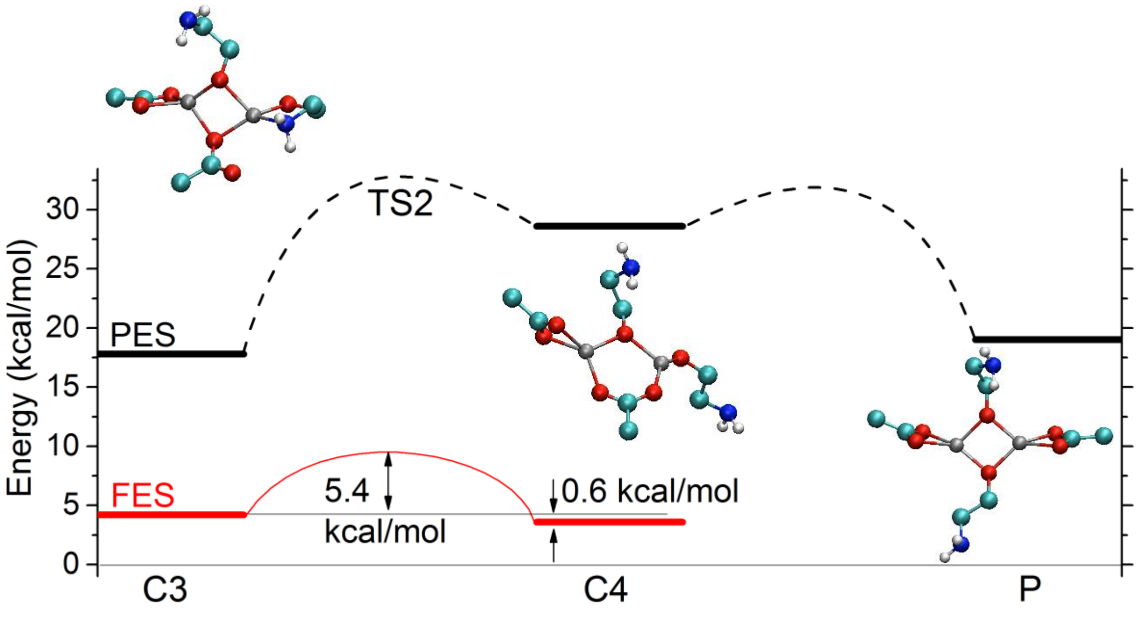

C4 **P**

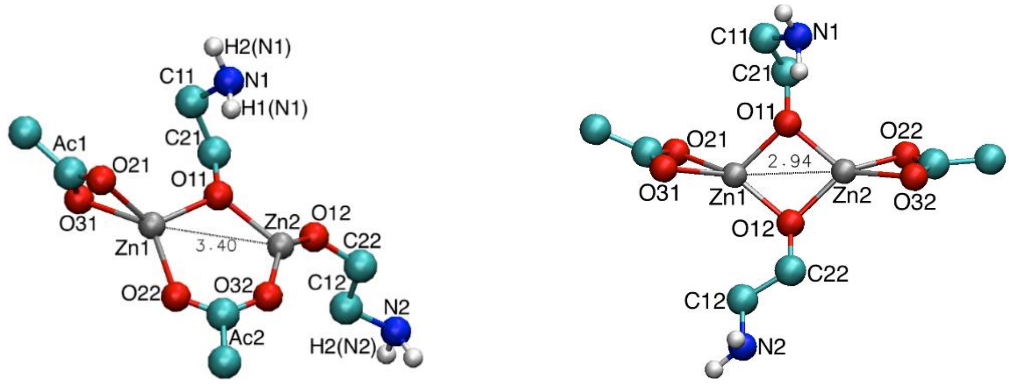

Figure S7. Free and potential energy profiles for the transformation **C3** → **C4,** and calculated energy for complex **P**.

Table S1. Electronic Kinetic Energies obtained for the systems $[Zn]_1$ and $[Zn]_2$ at different temperatures.

T (K)	300	570	670	770
$[Zn]_1$	0.005	0.009	0.010	0.011
$[Zn]_2$	0.010	0.021	0.024	0.027

Table S2. Final atomic coordinates for the optimized geometry of the dimer and calculated charges.

LABELS	SPECIES	COORDINATES (a.u.)			CHARGES (e)	
	$[Zn]_2$	X	Y	Z	INT	ESP
**C22**	C	22.5818	12.1549	15.1769	-0.025034	-0.094586
**Ac1**	C	15.0521	17.8446	11.0503	0.151537	1.041938
**C12**	C	21.0534	13.4083	17.3254	0.003501	-0.357987
**Ac1**	C	15.3126	19.9803	9155	-0.082513	-0.868023
**C21**	C	12.2659	15.7117	20.1963	-0.017129	-0.01456
**Ac2**	C	13.0302	10.312	12.0931	0.153412	1.049594
**C11**	C	9.9806	17.285	19.3335	0.007668	-0.230169
**Ac2**	C	11.6845	8.1157	10.8304	-0.083654	-0.76633
**H(EA2)**	H	21.2054	15.4807	17.1634	0.033072	0.220395
**Ac1**	H	15.8529	19.2038	7.3036	0.036537	0.248736
**H(EA2)**	H	24.5612	12.8472	15.3185	-0.003041	0.126038
**Ac1**	H	16.8597	21.2326	9.7638	0.037998	0.275051
**H(EA2)**	H	21.778	12.8305	19.199	0.012856	0.20023
**H2(N2)**	H	17.0586	13.8943	17.9813	0.040346	0.355399
**H(EA2)**	H	22.656	10.0746	15.5732	-0.010422	0.109272
**Ac1**	H	13.5577	21.0725	8.9943	0.020874	0.244423
**H1(N2)**	H	18.0226	10.9176	17.5553	0.099239	0.311351
**H(EA1)**	H	11.5651	13.8227	20.8359	-0.000432	0.07861
**H2(N1)**	H	7.8978	14.6391	17.2635	0.109693	0.206512
**H(EA1)**	H	13.0809	16.6439	21.8901	0.008751	0.13104
**Ac2**	H	10.4405	7.1605	12.1945	0.028529	0.223186
**Ac2**	H	10.473	8.8595	9.3079	0.036713	0.245375
**H(EA1)**	H	8.5117	17.3585	20.8157	0.020228	0.114638
**H1(N1)**	H	7.8765	17.4821	15.9783	0.114944	0.208699
**H(EA1)**	H	10.6114	19.2262	18.9201	0.03702	0.18547
**Ac2**	H	13.0442	6.7752	10.0234	0.028179	0.218198
**O31**	O	16.9003	16.3406	11.2529	-0.097373	-0.713291
**O12**	O	21.6017	12.6676	12.7701	-0.27691	-0.543404
**O21**	O	12.982	17.7525	12.3134	-0.160085	-0.714753
**O11**	O	14.0928	15.4978	18.2802	-0.210673	-0.644981
**O22**	O	11.6408	11.7703	13.4379	-0.149739	-0.774225
**O32**	O	15.3863	10.5293	11.7058	-0.11663	-0.726064
**Zn2**	Zn	17.9944	13.0382	13.0145	0.234683	0.833794
**Zn1**	Zn	12.4416	15.1465	14.9822	0.224819	0.650438
**N2**	N	18.3411	12.7529	17.005	-0.109245	-0.603628
**N1**	N	8.9918	16.2066	16.9185	-0.096062	-0.226385

Table S3. Final atomic coordinates for the optimized geometry of complex **C1** and calculated charges.

LABELS	SPECIES	COORDINATES (a.u.)			CHARGES (e)	
	C1	X	Y	Z	INT	ESP
**C22**	C	13.3372	10.661	9.7057	-0.020192	0.182267
**Ac1**	C	12.5744	14.3262	19.1467	0.148046	0.883735
**C12**	C	12.9275	13.54	9.8383	0.006057	-0.21994
**Ac1**	C	10.5789	13.7614	21.1165	-0.078577	-0.836316
**C21**	C	13.313	21.4724	14.386	-0.009055	-0.289865
**Ac2**	C	20.0764	13.4812	16.7542	0.163205	0.930138
**C11**	C	11.6963	22.0026	12.0362	-0.014671	0.188967
**Ac2**	C	22.7448	12.8825	17.5927	-0.078518	-0.914127
**H(EA2)**	H	11.1594	13.9433	10.8656	0.029144	0.155632
**Ac1**	H	10.1283	11.7389	21.154	0.034708	0.263519
**H(EA2)**	H	11.6546	9.8222	8.7732	0.004514	0.065912
**Ac1**	H	8.8534	14.8197	20.62	0.042273	0.237137
**H(EA2)**	H	12.7872	14.3663	7.9245	0.022308	0.099026
**H2(N2)**	H	14.6646	16.5863	11.809	0.043932	0.310292
**H(EA2)**	H	14.9723	10.3002	8.4143	0.001121	0.07506
**Ac1**	H	11.1947	14.4234	22.9867	0.031456	0.280612
**H1(N2)**	H	16.6548	14.7118	10.2578	0.11093	0.336588
**H(EA1)**	H	12.0334	21.0178	15.9887	0.010917	0.126114
**H2(N1)**	H	8.7411	23.845	13.8008	0.049539	0.331853
**H(EA1)**	H	14.3422	23.2273	14.9112	0.000632	0.173209
**Ac2**	H	23.0119	10.8375	17.7959	0.03259	0.288482
**Ac2**	H	24.0699	13.5959	16.153	0.04286	0.264603
**H(EA1)**	H	12.9623	22.3897	10.424	0.013936	0.133515
**H1(N1)**	H	10.8536	25.7723	12.5878	0.063722	0.3482
**H(EA1)**	H	10.623	20.2773	11.552	-0.001354	0.028345
**Ac2**	H	23.178	13.8653	19.37	0.035657	0.305963
**O31**	O	12.9583	12.7359	17.4023	-0.062994	-0.602358
**O12**	O	13.702	9.5404	12.0878	-0.278453	-0.680039
**O21**	O	13.7116	16.4692	19.3702	-0.171358	-0.655943
**O11**	O	15.0771	19.4811	13.8803	-0.250437	-0.599793
**O22**	O	19.4004	15.8031	16.9013	-0.143527	-0.651985
**O32**	O	18.7395	11.6643	15.9269	-0.121045	-0.636527
**Zn2**	Zn	15.3256	11.8857	14.2856	0.230497	0.776228
**Zn1**	Zn	15.976	17.265	16.518	0.29483	0.810072
**N2**	N	15.0236	14.7099	11.3171	-0.075163	-0.646845
**N1**	N	9.8997	24.1101	12.2627	-0.105347	-0.861732

Table S4. Final atomic coordinates for the optimized geometry of complex **C2** and calculated charges.

LABELS	SPECIES	COORDINATES (a.u.)			CHARGES (e)	
	C2	X	Y	Z	INT	ESP
**C22**	C	15.9793	13.4981	7.0169	-0.022847	-0.094859
**Ac1**	C	11.5476	15.783	21.5152	0.075151	0.904142
**C12**	C	14.2082	15.5839	8.0069	0.007367	-0.150819
**Ac1**	C	11.2799	16.0013	24.3442	-0.078502	-1.187966
**C21**	C	8.4273	18.3922	13.8585	-0.006752	-0.474787
**Ac2**	C	16.823	12.4629	16.7366	0.135583	0.854651
**C11**	C	8.6363	20.7393	12.182	-0.011552	0.130614
**Ac2**	C	17.2915	12.2911	19.5255	-0.063993	-0.989109
**H(EA2)**	H	12.2693	14.8244	8.1184	0.033778	0.131754
**Ac1**	H	9.7678	17.3731	24.7592	0.043223	0.353386
**H(EA2)**	H	15.2268	12.8378	5.1751	0.008544	0.07892
**Ac1**	H	13.0333	16.7206	25.1898	0.029561	0.340046
**H(EA2)**	H	14.2021	17.2366	6.7288	0.022673	0.108896
**H2(N2)**	H	13.3655	16.8664	11.7026	0.04381	0.303374
**H(EA2)**	H	17.8733	14.3458	6.6256	0.002509	0.142037
**Ac1**	H	10.7391	14.1796	25.1756	0.030256	0.305517
**H1(N2)**	H	16.2178	17.7844	10.6149	0.108159	0.35213
**H(EA1)**	H	6.8282	17.2089	13.1849	0.004527	0.173978
**H2(N1)**	H	10.5286	19.1245	9.2098	0.032762	0.327903
**H(EA1)**	H	7.9435	19.0227	15.7992	0.020208	0.179491
**Ac2**	H	19.1048	11.3587	19.896	0.036453	0.344065
**Ac2**	H	17.3317	14.2189	20.3209	0.044927	0.311439
**H(EA1)**	H	6.8996	21.8748	12.4229	0.017214	0.122891
**H1(N1)**	H	7.4788	19.3375	8.7501	0.051501	0.347131
**H(EA1)**	H	10.2215	21.9138	12.8678	0.010136	0.119907
**Ac2**	H	15.7339	11.2746	20.4543	0.046882	0.266604
**O31**	O	10.2334	14.1279	20.3158	-0.146755	-0.472072
**O12**	O	16.1615	11.4339	8.7058	-0.275042	-0.447731
**O21**	O	13.0344	17.2563	20.3174	-0.126537	-0.57776
**O11**	O	10.7219	16.9315	13.8235	-0.236301	-0.271421
**O22**	O	14.5646	12.916	15.8856	-0.098334	-0.407958
**O32**	O	18.5929	12.1536	15.1473	-0.111203	-0.536208
**Zn2**	Zn	16.3022	12.8121	12.0133	0.352127	0.549697
**Zn1**	Zn	11.6868	15.4048	16.9161	0.252287	0.353894
**N2**	N	14.9452	16.3204	10.6311	-0.118893	-0.628574
**N1**	N	9.0078	20.301	9.4632	-0.110907	-0.863203

Table S5. Final atomic coordinates for the optimized geometry of complex **C3** and calculated charges.

LABELS	SPECIES	COORDINATES (a.u.)			CHARGES (e)	
	C3	X	Y	Z	INT	ESP
**C22**	C	16.3293	11.6471	7.8066	-0.020783	-0.026533
**Ac1**	C	12.3517	15.0128	21.3433	0.077967	0.847738
**C12**	C	17.488	14.1974	6.9847	0.005174	-0.38596
**Ac1**	C	11.1399	14.7692	23.9083	-0.073264	-1.039269
**C21**	C	11.5617	16.9177	12.5568	0.016414	-0.197338
**Ac2**	C	20.1326	14.5758	16.3323	0.135066	1.022253
**C11**	C	9.742	18.6875	13.9308	0.001935	0.114213
**Ac2**	C	20.9852	14.8536	19.0505	-0.071567	-1.00535
**H(EA2)**	H	15.9605	15.5736	6.6352	0.030255	0.188901
**Ac1**	H	10.4594	16.63	24.5411	0.036129	0.328237
**H(EA2)**	H	14.9815	11.0655	6.3049	0.00285	0.089582
**Ac1**	H	12.5735	14.1409	25.2821	0.038851	0.29358
**H(EA2)**	H	18.6157	13.9921	5.2381	0.018117	0.154871
**H2(N2)**	H	19.5405	17.059	8.8617	0.114118	0.332461
**H(EA2)**	H	17.8777	10.2035	7.801	-0.002396	0.131141
**Ac1**	H	9.5823	13.4007	23.8652	0.030807	0.305728
**H1(N2)**	H	20.6455	14.1906	9.3964	0.097855	0.36397
**H(EA1)**	H	11.8668	17.6132	10.6045	0.015886	0.137307
**H2(N1)**	H	12.2365	21.5414	14.7432	0.055967	0.364147
**H(EA1)**	H	10.7338	15.0028	12.4094	0.033904	0.127009
**Ac2**	H	22.8531	15.7556	19.1181	0.026522	0.319767
**Ac2**	H	19.6109	15.9338	20.1798	0.043259	0.275838
**H(EA1)**	H	7.8594	18.523	13.036	0.022123	0.077012
**H1(N1)**	H	10.5278	22.0827	12.2212	0.052073	0.350869
**H(EA1)**	H	9.5058	18.0153	15.9009	0.023848	0.097593
**Ac2**	H	21.1272	12.9588	19.9053	0.043764	0.285864
**O31**	O	11.8244	13.4533	19.5756	-0.12122	-0.530196
**O12**	O	15.0979	11.7585	10.1583	-0.271067	-0.496604
**O21**	O	13.9392	16.8216	20.9424	-0.147329	-0.609403
**O11**	O	14.0039	16.8266	13.7777	-0.213974	-0.52484
**O22**	O	17.6729	14.0815	16.002	-0.156263	-0.603738
**O32**	O	21.5838	14.72	14.5134	-0.108517	-0.676446
**Zn2**	Zn	16.6768	14.2782	12.2469	0.235631	0.546413
**Zn1**	Zn	14.2914	15.598	17.252	0.245312	0.670907
**N2**	N	19.0126	15.2135	9.126	-0.107955	-0.499827
**N1**	N	10.4414	21.3743	14.0264	-0.10684	-0.829898

Table S6. Final atomic coordinates for the optimized geometry of complex **C4** and calculated charges.

**LABELS**	**SPECIES**	**COORDINATES (a.u.)**			**CHARGES (e)**	
	**C4**	**X**	**Y**	**Z**	**INT**	**ESP**
**C22**	C	14.1155	19.6107	7.9841	-0.004851	0.153323
**Ac1**	C	14.5761	9.4614	19.8558	0.073955	1.029617
**C12**	C	15.979	18.5872	6.0089	-0.010961	-0.048117
**Ac1**	C	13.3968	7.234	21.1949	-0.074092	-0.972091
**C21**	C	11.3128	13.4936	14.0259	0.027897	-0.266914
**Ac2**	C	18.8882	17.7988	16.8711	0.168178	0.861021
**C11**	C	9.4515	12.5555	15.9989	0.002255	-0.038922
**Ac2**	C	21.1652	19.1444	17.956	-0.079941	-0.947983
**H(EA2)**	H	15.1889	16.8115	5.2459	0.013634	0.126586
**Ac1**	H	14.2368	7.0183	23.083	0.036987	0.329257
**H(EA2)**	H	14.8867	21.3847	8.7947	0.009639	0.099755
**Ac1**	H	13.6122	5.486	20.1015	0.025629	0.230026
**H(EA2)**	H	17.7941	18.0953	6.954	-0.000682	0.094443
**H2(N2)**	H	17.2453	19.5848	2.4517	0.055941	0.359666
**H(EA2)**	H	12.3464	20.1291	6.9866	-0.000827	0.111335
**Ac1**	H	11.3652	7.6102	21.4726	0.043617	0.275764
**H1(N2)**	H	17.292	21.9406	4.4556	0.056784	0.391844
**H(EA1)**	H	10.3423	14.7411	12.6644	0.02452	0.216131
**H2(N1)**	H	6.9575	14.0489	18.5903	0.057444	0.342125
**H(EA1)**	H	12.1133	11.8742	12.9759	0.035643	0.122221
**Ac2**	H	21.4205	18.5844	19.9425	0.037234	0.282904
**Ac2**	H	22.8605	18.4976	16.9256	0.049354	0.303256
**H(EA1)**	H	7.9911	11.416	15.0343	0.027972	0.167333
**H1(N1)**	H	9.5595	15.7078	18.2532	0.055227	0.344984
**H(EA1)**	H	10.4638	11.2459	17.2952	-0.002528	0.055694
**Ac2**	H	21.0067	21.2039	17.7788	0.027408	0.29361
**O31**	O	15.4243	9.2106	17.5986	-0.136102	-0.61275
**O12**	O	13.5297	17.7571	9.8618	-0.277134	-0.630546
**O21**	O	14.6362	11.6288	20.9395	-0.137096	-0.709
**O11**	O	13.3429	14.9355	15.2048	-0.25679	-0.113351
**O22**	O	17.3898	19.0869	15.4713	-0.140446	-0.52298
**O32**	O	18.6993	15.4615	17.438	-0.138365	-0.543127
**Zn2**	Zn	14.6781	17.83	13.1756	0.378841	0.36258
**Zn1**	Zn	15.7345	13.1688	17.458	0.268392	0.624466
**N2**	N	16.2411	20.4046	3.8959	-0.103603	-0.94827
**N1**	N	8.2208	14.6979	17.2697	-0.101852	-0.82389

Table S7. Final atomic coordinates for the optimized geometry of the final **P** complex and calculated charges.

LABELS	SPECIES	COORDINATES (a.u.)			CHARGES (e)	
	P	X	Y	Z	INT	ESP
**C22**	C	15.632	12.744	19.3326	0.025274	-0.35321
**Ac1**	C	14.1601	16.329	10.5869	0.074363	0.908849
**C12**	C	14.5308	10.7185	17.597	0.004121	0.11843
**Ac1**	C	13.0369	16.6675	7.9897	-0.075033	-1.029009
**C21**	C	18.4513	21.5111	15.0767	0.027384	-0.47506
**Ac2**	C	21.4637	18.7737	22.8518	0.076092	0.911862
**C11**	C	16.4527	23.4946	14.4912	-0.004455	0.014977
**Ac2**	C	23.1445	19.4962	25.0348	-0.074077	-1.088458
**H(EA2)**	H	16.0948	9.6386	16.7414	0.026366	0.136833
**Ac1**	H	11.4241	15.3803	7.7375	0.035706	0.294111
**H(EA2)**	H	14.0739	13.6964	20.3628	0.025544	0.208554
**Ac1**	H	14.4546	16.3525	6.5108	0.028337	0.284778
**H(EA2)**	H	13.5111	11.6778	16.0231	0.01408	0.047656
**H2(N2)**	H	12.4959	7.4158	18.0132	0.062878	0.333416
**H(EA2)**	H	16.8661	11.8447	20.7554	0.023635	0.227212
**Ac1**	H	12.2978	18.6088	7.8338	0.039741	0.303767
**H1(N2)**	H	11.2799	9.8166	19.5579	0.06008	0.374363
**H(EA1)**	H	19.9287	22.3629	16.2815	0.024123	0.251524
**H2(N1)**	H	14.1564	25.9814	16.4304	0.056427	0.349542
**H(EA1)**	H	19.3394	20.8618	13.2991	0.028759	0.159004
**Ac2**	H	22.3285	21.1076	26.0609	0.035366	0.324758
**Ac2**	H	23.2442	17.8949	26.3636	0.041152	0.310765
**H(EA1)**	H	17.3488	24.9939	13.3462	0.019699	0.164834
**H1(N1)**	H	14.5909	23.273	17.8801	0.059901	0.326728
**H(EA1)**	H	14.9648	22.6103	13.2975	0.008889	0.034901
**Ac2**	H	25.0584	19.9429	24.3739	0.029299	0.33189
**O31**	O	16.5522	16.0469	10.8585	-0.132849	-0.538525
**O12**	O	17.0978	14.5469	17.9199	-0.23077	-0.250231
**O21**	O	12.7141	16.3872	12.5338	-0.137761	-0.577279
**O11**	O	17.3474	19.386	16.395	-0.239958	0.083809
**O22**	O	22.4225	18.2273	20.6994	-0.130202	-0.539953
**O32**	O	19.0534	18.6844	23.1679	-0.143211	-0.570195
**Zn2**	Zn	18.7071	17.6365	19.4024	0.289605	0.295774
**Zn1**	Zn	15.9949	16.2358	14.7763	0.259712	0.354195
**N2**	N	12.9571	8.9685	19.0755	-0.100734	-0.889601
**N1**	N	15.4948	24.6388	16.8381	-0.10517	-0.841011
